# Claudin-2 inhibits renal clear cell carcinoma progression by inhibiting YAP-activation

**DOI:** 10.1186/s13046-021-01870-5

**Published:** 2021-02-23

**Authors:** Balawant Kumar, Rizwan Ahmad, Giovanna A. Giannico, Roy Zent, Geoffrey A. Talmon, Raymond C. Harris, Peter E. Clark, Vinata Lokeshwar, Punita Dhawan, Amar B. Singh

**Affiliations:** 1 Department of Biochemistry and Molecular Biology, University of Nebraska Medical Center, 985870 Nebraska Medical Center, Omaha, NE 68198-5870 USA; 2 Department of Pathology, Microbiology and Immunology, Vanderbilt Medical Center, Nashville, TN USA; 3 Department of Medicine, Vanderbilt Medical Center, Nashville, TN USA; 4 Department of Pathology and Microbiology, University of Nebraska Medical Center, Omaha, NE 68198 USA; 5 Levine Cancer Institute, Charlotte, NC USA; 6 Department of Biochemistry and Molecular Biology, Augusta University, Augusta, GA USA; 7 Member, Fred and Pamela Buffett Cancer Center, University of Nebraska Medical Center, 985870 Nebraska Medical Center, Omaha, NE 68198-5870 USA; 8 VA Nebraska-Western Iowa Health Care System, Omaha, NE USA

**Keywords:** De-differentiation, Hippo/yap signaling, Renal Cancer, Proliferation

## Abstract

**Background:**

Claudin-2 expression is upregulated in multiple cancers and promotes cancer malignancy. Remarkably, the regulation of claudin-2 expression in kidney cell lines contrasts its reported regulation in other organs. However, claudin-2 role in renal clear cell carcinoma (RCC) remains unknown despite its predominant expression in the proximal tubular epithelium (PTE), the site of RCC origin.

**Methods:**

Publicly available and independent patient databases were examined for claudin-2 association with RCC. The novel protein function was validated in vitro and in vivo by gain or loss of function assays. Mechanistic results were concluded by Mass spectroscopy, immunoprecipitation and mutational studies, and functional evaluations.

**Results:**

We show that the significant decrease in claudin-2 expression characterized PTE cells and Ex-vivo cultured mouse kidney subjected to dedifferentiation. Inhibition of claudin-2 was enough to induce mesenchymal plasticity and invasive mobility in these models. Further, a progressive loss of claudin-2 expression associated with the RCC progression and poor patient survival. Overexpression of claudin-2 in RCC-derived cancer cells inhibited tumorigenic abilities and xenograft tumor growth. These data supported a novel tumor-suppressive role of claudin-2 in RCC. Mechanistic insights further revealed that claudin-2 associates with YAP-protein and modulates its phosphorylation (S127) and nuclear expression. The tumor suppressive effects of claudin-2 expression were lost upon deletion of its PDZ-binding motif emphasizing the critical role of the PDZ-domain in claudin-2 interaction with YAP in regulating RCC malignancy.

**Conclusions:**

Our results demonstrate a novel kidney specific tumor suppressive role for claudin-2 protein and further demonstrate that claudin-2 co-operates with the YAP signaling in regulating the RCC malignancy.

**Supplementary Information:**

The online version contains supplementary material available at 10.1186/s13046-021-01870-5.

## Background

The renal clear cell carcinoma (RCC) is the most common histological subtype, and malignant, of the renal cancers that represents 70 to 80% of all renal cancer cases [1]. Unfortunately, at the time of the initial diagnosis about 30% of patients have metastatic disease and another ~ 30% develop metastasis despite nephrectomy [2]. The treatment of metastatic RCC is problematic as despite emergence of the novel targeted therapies over the last decade, survival rates remains less than 10% [3]. This grim prognosis poses the need for an improved understanding of the underlying molecular mechanisms driving metastatic RCC, to be able to develop novel therapeutic approaches.

A historical perspective suggests that deregulation of the epithelial cell-cell contact and polarity programs, known features of the cancer cells, facilitate rewiring of the tumor suppressive and oncogenic signaling events, to promote malignant growth and metastasis [4]. In this regard, tight junctions, the most apical cell-cell adhesion, play important role in maintaining the cell-cell contact and epithelial cell polarity. The claudin family of proteins constitute the bulwark of the tight junction and has been widely implicated in preserving the epithelial cell polarity and differentiation [5]. Accordingly, dysregulation of specific claudin proteins is associated with neoplastic growth, albeit in a tissue-specific manner [6–8]. Among claudin proteins, claudin-2 is a cation-selective isoform, which is highly expressed in the proximal tubular epithelium (PTE). In this regard, studies have now validated critical role of claudin-2 in renal Na ^+^ and Ca^++^ homeostasis [9]. However, claudin-2 knockout mice are viable though demonstrate increased renal transcellular Na ^+^ −transport and oxygen consumption [10]. Claudin-2 deficiency also associates with the renal hypercalciuria in mice and human renal stone disease [11]. The proximal tubular epithelium is also the prime source for the RCC [12]. However, potential role of claudin-2 in RCC remain unknown despite the fact that published studies from our and other laboratories have demonstrated that in comparison to other claudin proteins, the expression of claudin-2 is rather sensitive to the differentiated status of proximal tubular epithelial (PTE) cells and to the signaling mechanisms that promote EMT [13, 14].

The Hippo-signaling pathway regulates cell proliferation and is regulated by multiple attributes of the physical state of cells in mammalian tissues [15]. The Hippo-pathway consists of a series of serine kinases where the Ser/Thr kinase Hippo in *Drosophila* (Mst 1 and Mst2 in mammals), ultimately regulates phosphorylation of the transcriptional activator YAP to regulate its nuclear localization [16]. The phosphorylation of YAP is often taken as a measure of the activity of the Hippo pathway [17]. Beyond, regulating the normal cellular homeostasis, the Hippo/YAP signaling exerts a critical role in modulating tumorigenic abilities of cancer cells and accordingly has been demonstrated to contribute to the progression of various cancers. It has been detected that the abnormal Hippo-signaling  pathway can result in hyperactive YAP function, including in kidney [18]. The nuclear overexpression of YAP has been observed in patients with RCC [19]. Related studies have further demonstrated causal role of the YAP-signaling in promoting RCC malignancy and therapeutic responses. However, molecular regulation of the YAP-signaling in RCC remains poorly understood.

In the present study, utilizing a combined in vitro and in vivo approach, including mouse models of renal tumorigenesis and RCC patient data, we have discovered a novel tumor suppressive role for claudin-2 in RCC. We further demonstrate that claudin-2 regulates YAP phosphorylation and nuclear localization to regulate proximal tubular epithelial transformation and oncogenic growth. Taken together, our data supports role of claudin-2 as a novel prognostic biomarker of RCC progression and a potential target candidate for the future treatment of the RCC.

## Methods

### Cell culture, transfection and reagents

MDCK-II, HK-2 and Caki-2 cells were either purchased from ATCC (American Type Culture Collection, Rockville, MD, USA) or were available in our laboratory, and have been described previously. Cell lines are routinely verified for authenticity by genomic analysis at UNMC core facility. MDCKII^TALEN^ (Claudin-2 knockout) cells were kindly provided by Dr. Mikio Furuse, Kobe University Graduate School of Medicine, Japan. The YAP mutant constructs were kind gift from Dr. Jixin Dong, University of Nebraska Medical center, Omaha, NE. Cells were maintained in Dulbecco’s Modified Eagle’s medium F12 (DMEM-F12) or McCoy’s 5A Medium, as appropriate, which were supplemented with 10% fetal bovine serum (FBS) and Penicillin-Streptomycin (50 μg/ml). Cell transfection was performed as described previously [20]. All reagents and antibodies used in this study are listed in the supplementary Table-S1.

### 3D ex-vivo kidney culture

For 3D Ex-vivo kidney culture, mice were sacrificed, kidneys were removed and immediately sliced (of identical thickness) using a sharp razor blade. All procedures were performed in sterile conditions at 4 °C. Resulting kidney slices were embedded in a Matrigel bed in 24-well culture dish and bathed in special cell culture medium containing N2 (50 μM) and B27(100 μM) as supplements, as done for the 3D-organ culture [21]. 3D-tissue culture medium contained DMEM/F12 and Penicillin-Streptomycin, and Kidney slices were cultured in above culture conditions for at least 48-h with or without experimental manipulations, and then subjected to the lysate preparation.

### Human renal cancer tissue collection and processing, microarray platforms and statistical analysis

The protocols and procedures for the procurement of human tissue samples have been described previously [22]. In brief, a TMA was constructed from the tumors of 207 patients who underwent radical nephrectomy between 1994 and 2006 with confirmed histologic diagnosis of ccRCC. Two punches from areas of histologically viable tumor without evidence for necrosis or hemorrhage and 2 punches from histologically normal renal cortex from each patient were embedded in the TMA block (828 cores total). Patient demographic, pathologic, and clinical outcomes data were extracted from the electronic medical records and anonymously linked to the TMA cores under an Institutional Review Board approved protocol. All cases were assigned a Fuhrman nuclear grade and staged in accordance with the 2010 American Joint Committee on Cancer (AJCC) guidelines. To compare the expression level of claudin-2 between the normal tissue and renal cancer tissues, the Mann–Whitney tests were conducted, and exact *P*-values were reported. In addition, analysis of the TCGA (The Cancer Genomic Atlas) database was done using the UALCAN software available online (http://ualcan.path.uab.edu/analysis.html).

### Mouse model of spontaneous renal tumorigenesis

The offspring mice γGT-Cre/Kras ^+/G12D^/Catnb^+/lox(ex3)^ (referred to as Kras^+G12D^/Catnb^Δex3^) produced by crossing the mice harboring γGT-Cre recombinase and mice with a conditional activating mutation of *Ctnnb1* (Catnb^lox(ex3)^) and *Kras* (LSL-Kras^G12D^) have been described previously [23].

### Statistical analysis

Student’s *t*-test, Fisher exact test and analysis of variance were used to determine statistical significance as applicable, and differences were considered statistically significant at *P* < 0.05. Results were plotted using Prism 9.0 (GraphPad Software, Inc.). All data presented are representative of at least three repeat experiments and are presented as mean ± sem unless described otherwise.

## Results

### A significant decrease in claudin-2 expression characterizes RCC progression and predicts poor patient survival

**Fig. 1:**
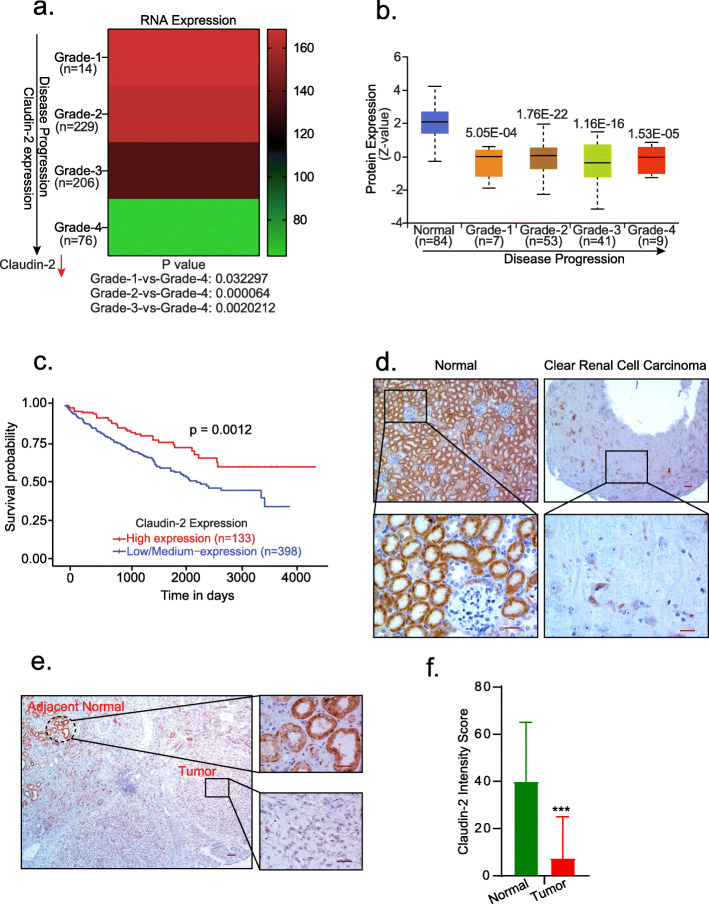
Claudin-2 expression is sharply downregulated in human renal clear cell carcinoma and associates with poor patient survival: (**a**) Claudin-2 transcriptome analysis in TCGA database [*P < 0.03 (stage-1Vs stage-4), ****P < 0.00006 (Stage-2 Vs stage-4,**P < 0.0020212 (Stage-3 Vs Stage-4) (**b**) Represent claudin-2 proteomic expression profile based on individual cancer stage of RCC in CPTAC (Clinical Proteomic Tumor Analysis Consortium) database (Normal Vs grade 1 *P* < 5.05E-4; Normal Vs grade 2 *P* < 1.76E-22; Normal Vs grade 3 *P* < 1.16E-16; Normal Vs grade 4 *P* < 1.53E-5); (**c)** Kaplan-Meir analysis to determine overall patient survival in relation with claudin-2 expression. The red and blue colors indicate high and low claudin-2 expression, respectively (***P < 0.0012); (**d**) Representative images of immunohistochemistry of claudin-2 using RCC patient’s TMA (*N* = 207); (**e**) Representative image of the immuno-histochemical analysis of claudin-2 expression in archived renal cancer surgical specimen with adjacent normal tissue; (**f**) Represent the intensity score of claudin-2 in patient samples. Data is presented as mean + sem. Statistical significance was determined by 1-way ANOVA and post hoc Tukey’s test for pairwise comparison. ***P < 0.001. Scale bar = 50 μM

To test a role for claudin-2 in RCC, we determined if claudin-2 expression is altered in the patient samples. We examined The Cancer Genome Atlas (TCGA) database using UALCAN (http://ualcan.path.uab.edu/index.html), an online database web portal. Interestingly, this analysis demonstrated a significant decrease in claudin-2 expression in RCC patient samples and also that this decrease was specific to the cancer stages/progression (Fig. 1a). Further analysis of protein expression using Clinical Proteomic Tumor Analysis Consortium (CPTAC) database revealed a similar progressive decrease in claudin-2 protein expression with RCC progression (Fig. 1b). We also found significant association of the low claudin-2 expression with poor patient survival, based on the overall survival, tumor grade or patient gender (Fig. 1c; Fig. S1 a and b). To further validate, we performed immunohistochemical (IHC) analysis of an independent RCC tissue microarray (TMA; 207 Samples) using anti-claudin-2 antibody. Robust claudin-2 expression in proximal tubules in the normal kidney supported IHC specificity. Claudin-2 immunoreactivity was sharply decreased in the renal carcinoma samples (Fig. 1d). Blinded analysis by a renal pathologist validated a significant and inverse association between claudin-2 expression and RCC. Additional inquiry using a panel of large surgically removed RCC specimen further verified a sharp decrease in claudin-2 expression in the tumor and adjacent fibrotic areas (Fig. 1e). Intensity scoring for claudin-2 expression in RCC patient samples by a pathologist further showed significant decreases in the tumor versus normal kidney (Fig. 1f). Taken together, above data provided strong evidence of an unexpected inverse association between claudin-2 expression and RCC progression.

### Claudin-2 expression is markedly downregulated in a spontaneous model of renal tumorigenesis

**Fig. 2:**
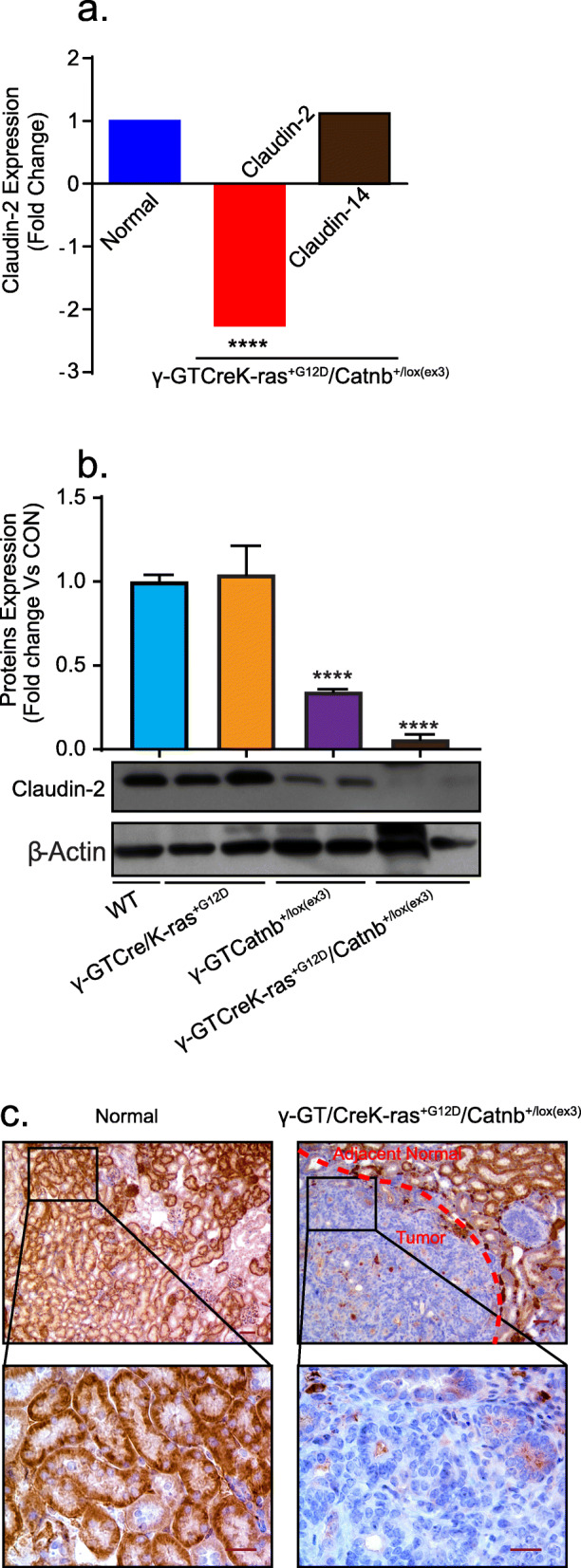
Claudin-2 expression is sharply downregulated in mouse model of spontaneous renal tumorigenesis: (**a**) Microarray analysis to determine mRNA expression changes in normal mouse kidney or KRas and β-catenin mutated mice kidney (γ-GTCreK-ras^+G12D^/Catnb+/^Lox(ex3)^ with spontaneous tumor growth. RNA was extracted from the kidney of three individual mice from each group (two from Kras^G12D^/Catnb^Δex3^). Data shown for claudin-2 and claudin-14 expression; (**b**) Representative immunoblot and densitometric analysis of renal claudin-2 expression in γ-GT^Cre^ (WT), γ-GT/CreK-ras^+G12D^ (activated K-ras in PTE cell), γ-GTCatnb^+/Lox(Δex3)^) (activated β-catenin in PTE cells) and γ-GT/CreK-ras^+G12D^/Catnb^+/Lox(Δex3)^ (activated K-ras and β-catenin in PTE cells) (*N* = 2/group, except wild type); (**c**) Representative images of immunohistochemical determination of claudin-2 expression in normal mice kidney or mice with spontaneous renal tumors (y-GT/CreK-ras^+G12D^/Catnb^+/Lox(Δex3)^. Data is presented as mean + sem. Statistical significance was determined by 1-way ANOVA and post hoc Tukey’s test for pairwise comparison. ****P < 0.0001. Scale bar = 50 μM

Based upon the above findings, we postulated a tumor suppressive role for claudin-2 in RCC. To test this hypothesis, we used mice that were genetically engineered to express activated K-ras and β-catenin in a PTE specific manner, as these mice develop spontaneous renal tumors (γGT-Cre/Kras^+/G12D^/ Catnb^+/lox(Δex3)^) [24, 25]. Remarkably, RNAseq data using the total RNA from the kidneys from Kras^G12D^/Catnb^Δex3^ mice demonstrated a significant decrease in claudin-2 (versus littermate Cre-mice kidney). In the same samples, expression of claudin-14 was not altered and thus verified the specificity of the decreases in claudin-2 expression (Fig. 2a), which we further confirmed at the protein level using total kidney lysates from these mice (Fig. 2b). IHC analysis of the kidney from these mice further demonstrated a sharp decrease in claudin-2 expression in the tumor with adjoining normal tissue demonstrating robust claudin-2 expression (Fig. 2c). The immunoblot analysis also revealed potential synergy between Wnt/β-catenin signaling and K-Ras mutation in regulating renal claudin-2 expression as it was markedly down in mice with constitutive Wnt/β-catenin signaling in renal epithelium however decreased to its nadir when combined with the K-Ras mutation (Fig. 2b). A role of Wnt-signaling in RCC progression is well recognized [26]. Overall, these data strongly supported a causal role of claudin-2 loss in promoting EMT and renal tumorigenesis.

### Induction of EMT in PTE cells specifically inhibits claudin-2 expression among claudin proteins

#### A) an inverse association between claudin-2 levels and EMT characterize MDCK-II cells

**Fig. 3:**
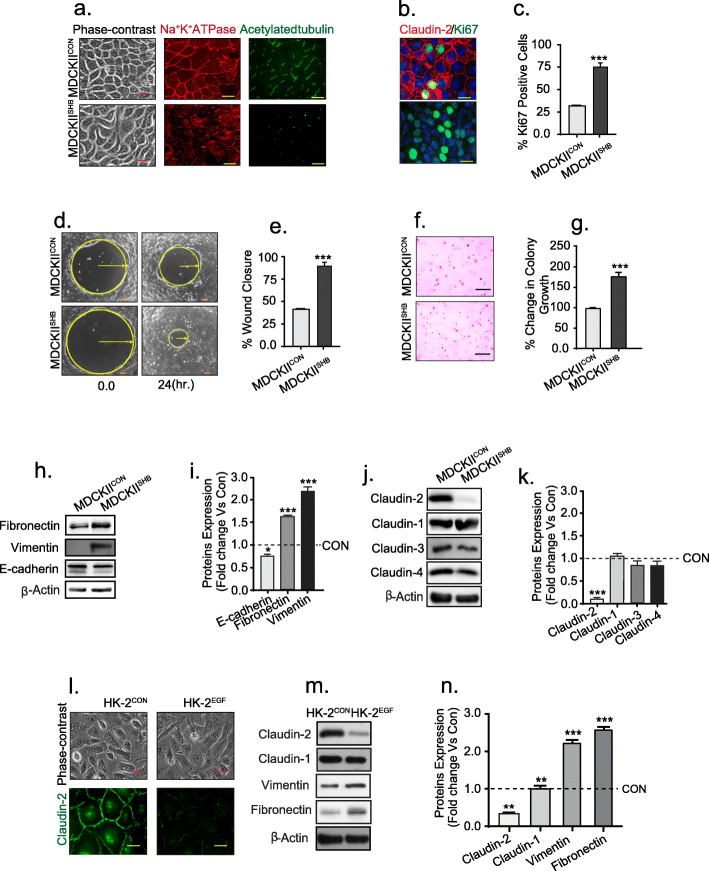
Stimulation of EMT in PTE cells specifically inhibits claudin-2 expression among claudin proteins: Cells stably overexpressing the empty cloning vector (MDCKII^CON^) or a mutant HB-EGF construct that is constitutively secreted into the medium (MDCKII^SHB^) were used. **a** Representative phase-contrast images, and immunofluorescent analysis of the Na ^+ ^K^+^ ATPase expression and primary cilium (acetylated-tubulin); (**b**-**c**) Immunofluorescent co-localization of Ki-67 and claudin-2 proteins, and quantitative analysis; (**d**-**e**) Representative images of MDCKII^CON^ and MDCKII^SHB^ cells subjected to wound-healing (time-course analysis), and quantitative analysis; (**f**-**g**) Anchorage-independent growth (colony formation assay) in soft agar, and its quantitative analysis; (**h**-**i**) Immunoblot analysis of EMT markers, and its densitometric analysis; (**j**-**k**) Immunoblot analysis of claudin proteins; Confluent HK-2 cells were subjected to EGF (100 ng/ml) treatment (**l**) Representative phase contrast images and immunolocalization of claudin-2 protein in control and EGF-treated cells; (**m**-**n**) Representative immunoblot and its densitometric analysis using total cell lysate from control and EGF-treated HK-2 cells;. Data is presented as mean + sem (scale bar =50 μM). Statistical significance was determined by student t test and 1-way ANOVA. *P < 0.05, **P < 0.01; ***P < 0.001

In light of above data, we further examined if EMT induction in PTE cells would downregulate claudin-2 expression. We first used MDCK-II cells, based on the fact that they are the best used model of epithelial cell polarity and differentiation, and because they bear PTE cell characteristics including robust claudin-2 expression [27, 28]. We used EGFR-overactivation to induce EMT based on its known role in promoting malignant growth, including in RCC [29]. We used MDCKII^SHB^ cells that express a mutant construct of heparin-binding EGF like growth factor (HB-EGF). This construct encodes a secreted form of HB-EGF, which in turn induces activation of EGF-receptor (EGFR) in an autocrine/paracrine manner [30]. Cells expressing empty vector served as control (MDCKII^CON^). MDCKII^SHB^ cells presented typical transformed mesenchymal cell phenotype and showed significant loss of the expression of membrane-tethered Na^+^K^+^ ATPase and of primary cilium (acetylated tubulin), used as markers of the differentiated PTE cells, as compared to the MDCKII^CON^ cells (Fig. 3a). Cell proliferation, migration and colony formation in soft agar were also significantly upregulated in MDCKII^SHB^ cells (MDCKII^SHB^ versus control cells; Fig. 3b-g*)*. Moreover, a significant increase in mesenchymal cell markers (vimentin and fibronectin) and a corresponding decrease in E-cadherin expression was observed in MDCKII^SHB^ cells (versus MDCKII^CON^ cells; Fig. 3h and i). Interestingly, among claudin proteins tested, only expression of claudin-2 was inhibited in MDCKII^SHB^ cells, though the membrane tethered expression of all claudin proteins was compromised (Fig. 3j and k*;* Fig. S2). Extended culture of the serum starved MDCK-II cells in the presence of EGF (100 ng/ml) produced similar results (Fig. S3 a-j).

To ascertain that the above findings are not limited to the chronic EGFR-stimulation, we subjected MDCK-II cells to chronic hypoxia [(CO_2_ (5%), Nitrogen (94%) and O_2_ (1%)]. Hypoxia promotes cancer progression and is reported to induce EMT in MDCK-II cells [31]. Morphological assessment as in above studies and expression of mesenchymal cell markers, supported development of EMT in MDCK-II cells subjected to persistent hypoxia (versus normoxia) (Fig. S4 a). Immunoblot analysis further demonstrated a time-dependent and sharp decrease in claudin-2 expression in hypoxia-challenged cells, which interestingly preceded the decreases in the E-cadherin. Fibronectin and vimentin expression were significantly upregulated while, claudin-1 levels remained largely unaltered in same samples (Fig. S4 b and c). Immunofluorescence analysis supported these data (Fig. S4 d).

#### B) an inverse association between claudin-2 levels and EMT characterize human kidney PTE (HK-2) cells

To determine if the observed effect of claudin-2 downregulation on the EMT phenotype was not limited just to MDCK-II cells, we assessed if HK-2 (human kidney PTE cells) behave in a similar fashion. Initial characterization confirmed robust levels of membrane localized claudin-2 expression in HK-2 cells (Fig. 3l). To induce EMT, HK-2 cells were treated either with EGF (100 ng/ml), TGF-β (10 ng/ml, data not shown) or chronic hypoxia. As in the case of MDCK-II cells, an inverse association was observed between claudin-2 expression and EMT in the HK-2 cells (Fig. 3l-n; Fig. S4 e-g). This indicates that induction of an EMT phenotype following claudin-2 downregulation occurs in PTE cells. Taken together, the above data suggested an unexpected and novel role of claudin-2 protein in maintaining the differentiated epithelial phenotype in PTE cells and contact inhibition of growth.

### Claudin-2 knockout promotes plasticity in renal epithelial cells in vitro and in vivo and precocious EMT

#### A) TALEN-induced claudin-2 knockdown in MDCK-II cells induces mesenchymal plasticity

**Fig. 4:**
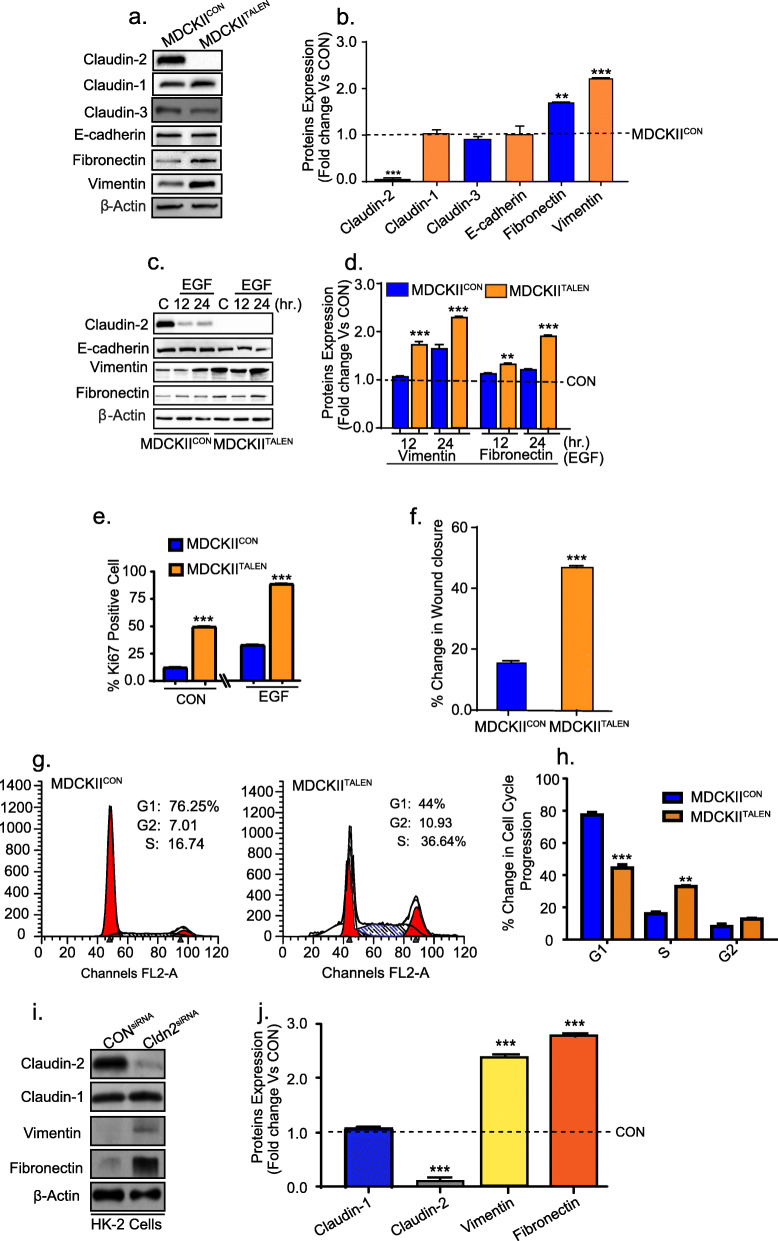
Claudin-2 knockdown in PTE cells increases plasticity and proliferation: MDCKII^TALEN^ cells where claudin-2 expression was knocked down by TALEN mediated genetic editing were subjected to the EMT analysis compared to the control MDCKII cells. **a**-**b** Immunoblot and its quantitation analysis using total cell lysates from MDCK-II and MDCKII^TALEN^ cells; (**c**-**d**) Immunoblotting and densitometric analysis using total cell lysates from MDCK-II and MDCKII^TALEN^ cells subjected to the EGF (100 ng/ml) treatment in time dependent manner; (**e**) Quantitative analysis of Ki-67 positive cells in MDCKII and MDCKII^TALEN^ cells treated with or without EGF; (**f**) Quantitative analysis of the wound-healing in MDCKII and MDCKII^TALEN^ cells; (**g**) Cell cycle analysis of MDCKII and MDCKII^TALEN^ cells by flow cytometry using PI staining. **h** The quantitative measurement of different cell cycle phases; (**i**-**j**) Representative immunoblots and quantitation of EMT related and claudin proteins in control HK-2 cells (CON^siRNA^) vs cells expressing anti-human claudin-2 siRNA (Cldn2^siRNA^). Data is presented as mean + sem. Statistical significance was determined by student t test and 1-way ANOVA and post hoc Tukey’s test for pairwise comparison. ** *P* < 0.01, ***P < 0.001

To verify above novel findings, we then examined if claudin-2 knockout in MDCK-II cells can induce similar plasticity and promote EMT. In this regard, we first used MDCKII^TALEN^ cells, in which claudin-2 gene expression was knocked out using the TALEN-mediated gene editing (Fig. S5 a) [32]. Immunoblot analysis confirmed that the MDCKII^TALEN^ cells are deficient in claudin-2 expression. Expression of other claudins was not majorly affected (Fig. 4a and b*)*. However, marked increases in the vimentin and fibronectin expression in MDCK^TALEN^ cells (versus MDCK-II^CON^ cells) validated the causal role of claudin-2 loss in promoting mesenchymal plasticity in PTE cells (Fig. 4a and b). While, total E-cadherin expression in MDCK^TALEN^ cells was not markedly altered, its membrane localization was compromised (Fig. S5 b). Further, when subjected to the EMT by EGF-stimulation or hypoxia, MACKII^TALEN^ cells demonstrated more EMT (versus MDCK-II^CON^ cells; Fig. 4c and d; Fig. S5 c). The EGF-induced proliferation and migration in MDCK^TALEN^ cells were also significantly higher compared to MDCK-II^CON^ cells (Fig. 4e and f; Fig. S5 d and e). Cell cycle analysis of the synchronized MDCKII and MDCKII^TALEN^ cells further demonstrated a significant increase in the number of cells in the S-phase in MDCKII^TALEN^ cells (versus MDCKII^CON^ cells; Fig. 4 g and h) and thus supported the overall findings.

#### B) siRNA-induced claudin-2 knockdown in HK-2 cells induces EMT

To further validate, we silenced claudin-2 expression in HK-2 cells using anti-human claudin-2 siRNA. Immunoblotting of the cell lysate prepared 48 h post-siRNA transfection confirmed significant inhibition of claudin-2 expression and concomitant increases in vimentin and fibronectin expression (versus HK-2 cells transfected with control siRNA; Fig. 4 i and j). In addition, we developed 3D Ex-vivo culture of kidney from wild type and claudin-2 knockout mice, as described in “Materials and Methods”. Kidney slices in this 3D Ex-vivo culture was subjected to hypoxia. As shown in Fig. S5 f and g, immunoblotting using total lysates prepared from the kidney slices grown in either normoxic or hypoxic culture conditions clearly demonstrated that the loss of claudin-2 expression promotes mesenchymal plasticity (increased fibronectin and vimentin expression) in PTE cells.

### Claudin-2 overexpression in RCC-derived cancer cells inhibits mesenchymal plasticity and tumorigenic abilities

**Fig. 5:**
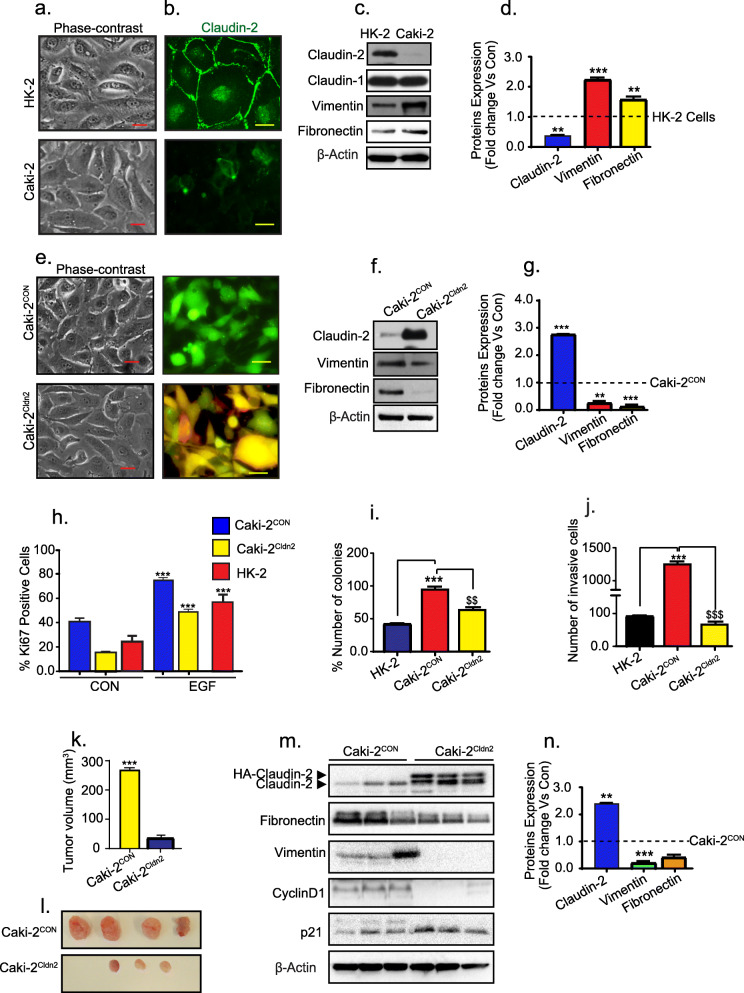
Claudin-2 overexpression in Caki-2 cells restores epithelial phenotype, contact inhibition of growth and inhibit tumorigenesis: The RCC-derived Caki-2 cells were stably transfected with full-length claudin-2 expression construct (Caki-2^Cldn2^) or cloning vector (caki-2^CON^). Effects on EMT and tumorigenic properties in vitro and in vivo were determined (**a**) Representative phase-contrast images, and (**b**) Immunofluorescent analysis of claudin-2 expression in HK-2 and Caki-2 cells; (**c** and **d**) Immunoblot analysis and quantitation using total cell lysates of HK-2 and Caki-2 cells; (**e**) Phase-contrast images and fluorescent images of the control (Caki-2^CON^) and claudin-2 overexpressing cells (Caki2^Cldn2^); (**f** and **g**) Representative immunoblots and densitometric analysis of EMT marker and claudin-2; (**h**) Quantitative analysis of Ki-67positive cells; (**i**) Anchorage-independent growth in the soft agar; (**j**) Relative cell invasion; (**k** and **l**) Represent effects of claudin-2 overexpression on tumor forming abilities of Caki-2 cells using a subcutaneous xenograft model in athymic nude mice, and representative images (n# 4/group) where one tumor did not grow to measureable size in Caki2^Cldn2^ injected mice; (**m** and **n**) Immunoblot analysis and quantitation using the total tumor tissue lysates from Caki-2^CON^ and Caki-2^Cldn2^ cells. Statistical significance was determined by student t test and 1-way ANOVA and post hoc Tukey’s test for pairwise comparison. ** *P* < 0.01 and ***P < 0.001 ($ = caki-2^CON^ vs Caki^cldn2^ (for invasion assay and colony formation assay). Scale bar = 50 μM

To validate further that claudin-2 serves a tumor-suppressive role in RCC, we utilized RCC-derived Caki-2 cells [33]. A comparative analysis of HK-2 and Caki-2 cells revealed minimal claudin-2 expression in Caki-2 cells (versus HK-2 cells) and contrasting higher expressions of vimentin and fibronectin (Fig. 5a-d). We stably overexpressed a full-length claudin-2 expression construct in Caki-2 cells, represented as Caki-2^Cldn2^ (see Supplementary methods and Supplementary Fig. S6 a-d) (Fig. 5e). Immunoblotting verified robust claudin-2 overexpression in Caki-2^Cldn2^ cells, which was also associated with marked downregulation of vimentin and fibronectin expression (versus control cells; Fig. 5f-g*)*. We also found significant decrease in Ki-67 positive cells, colony formation in soft agar and cell invasion in Caki-2^Cldn2^ cells versus control Caki-2 cells or HK2 cell (Fig. 5h-j; Fig. S6e-g). To further assess the effects on in vivo tumorigenesis, we performed xenograft tumor implantation in athymic nude mice using Caki-2^CON^ and Caki-2^Cldn2^ cells, as described in the “Supplementary methods”. As shown in Fig. 5k and l, claudin-2 overexpression resulted in striking inhibition of the tumor formation and growth by Caki-2 cells (290 ± 1.2 mm^3^ versus 3 ± 0.6 mm^3)^, at the time of mice sacrifice (3weeks from the time of subcutaneous injection). Immunoblot analysis using the lysate prepared from these xenograft tumors affirmed sustained claudin-2 expression in the tumors resulting from the use of Caki-2^Cldn2^ cells along with inhibition of vimentin and fibronectin and cyclin-D1 expression. In the same samples, we found contrasting increases in P-21 expressions compared to the tumors resulting from the use of Caki-2^CON^ cells (Fig. 5m and n). Taken together, these data validated a tumor suppressive role for claudin-2 in RCC.

### Claudin-2 associates with YES-associated protein (YAP) and modulates its activation to regulate PTE cell plasticity and RCC tumorigenesis

**Fig. 6:**
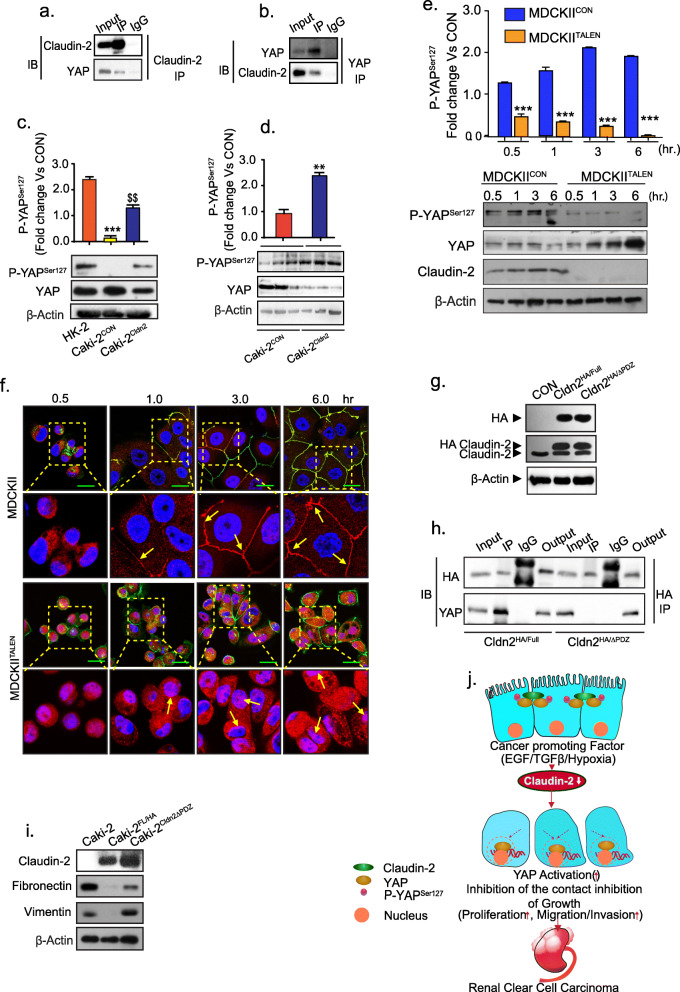
Claudin-2 interacts with YAP through its PDZ binding motif and hinders its nuclear localization and activation. **a** Total cell lysate from MDCK-II cells was subjected to immunoprecipitation using anti-claudin-2 antibody and then immunoblotted with anti-YAP; (**b**) Total cell lysate from MDCK-II cells was subjected to immunoprecipitation using anti-YAP antibody and immunoblotting with anti-claudin-2 or anti-YAP antibody; (**c**) Immunoblotting of p-YAP^S127^/YAP and its densitometry analysis; (**d**) Immunoblot analysis and quantitation of p-YAP^S127^/YAP using the total tissue lysates from Caki-2^CON^ and Caki-2^Cldn2^ cell induced (xenograft) tumors; (**e**) Representative immunoblot analysis of p-YAP^S127^, YAP and claudin-2 expression using total cell lysate from MDCKII and MDCKII^TALEN^ cells subjected to a time-course of growth, and densitometric quantitation of p-YAP^S127^; (**f**) Immunofluorescent analysis of YAP and ZO-1 proteins in MDCKII and MDCKII^TALEN^ cells growing in time dependent manner (30 min to 6 h). Yellow box area from MDCKII cells and MDCKII^TALEN^ cells was enlarged below to show YAP localization. Arrows indicate localization of the YAP protein; (**g**) Immunoblot analysis of MDCK-II cell lysate expressing claudin-2-HA recombinant protein (expressing Cldn2^FL/HA^ plasmid) or claudin-2 protein lacking the PDZ-binding motif (expressing Cldn2^ΔPDZ/HA^ plasmid); (**h**) Immunoprecipitation using anti-HA antibody. Total cell lysate of MDCK-II cell expressing Cldn2^FL/HA^ plasmid construct and Cldn2^ΔPDZ/HA^ construct was used for immunoprecipitation and subsequently immunoblotted with anti-HA or anti-YAP antibody; (**i**) Representative Immunoblots of EMT marker in Caki-2 cells transfected with Cldn2^FL/HA^ or Cldn2^ΔPDZ/HA^ expression construct; (**j**) Graphical modelling of the overall concept. Differentiated PTE cell having apical claudin-2 (green) which interacts with YAP (brown) protein and sequestered to membrane. Cancer promoting factors induce down-regulation of claudin-2 expression. Loss of claudin-2, in turn, promotes loss of cell-cell contact and de-localization of YAP protein to the cell nucleus and hence induction of proliferation and RCC progression. Data is presented as mean + sem. Statistical significance was determined by student t test and 1-way ANOVA and post hoc Tukey’s test for pairwise comparison. ** or $$ *P* < 0.01; ***P < 0.001

To determine further how claudin-2 protein affects PTE cell plasticity and RCC progression, we examined potential protein interactions with claudin-2 in our model system. Of interest, claudin-2 is part of a large protein complex and was recently shown to interact proteins that regulate cell polarity and cell-cell adhesion [34, 35]. High throughput proteomics analysis was done using MDCK-II and MDCK-II^TALEN^ cells as detailed in “Material and Methods”*.* Mass spectrometry analysis identified several proteins (~ 110 proteins) that co-immunoprecipitated with claudin-2 in MDCK-II cells (and absent in the immunoprecipitate using MDCKII^TALEN^ cell lysate) including caveolin-1, ZO-1, ZO-3, Ubiquitin carboxyl-terminal hydrolase etc. that are reported to interact with claudin-2 [36, 37] (Fig. S7 a and Supplementary Table-S3). Interestingly, YAP (Yes associated Protein), a key component of the Hippo/YAP signaling, also co-immunoprecipitated with claudin-2. A role for YAP-activation in promoting RCC has been demonstrated [19, 38]. Of interest, Hippo/YAP signaling is regulated by cell-cell contact inhibition, which prevents nuclear localization of YAP and transcriptional activity [15]. We validated this finding in co-immunoprecipitation studies using anti-claudin-2 or YAP-antibody for the pull-down assay followed by immunoblotting with the respective other antibody (Fig. 6 a and b). Published studies of others, have demonstrated that claudin-2 expression increases in PTE cells with contact inhibition and differentiation [39]. Moreover, immunoblot analysis using lysates from Caki-2^CON^ and Caki-2^Cldn2^ cells demonstrated a positive association between claudin-2 expression and phospho-YAP^Ser127^ expression (inactive YAP). HK-2 cells served as control (Fig. 6c). Furthermore, we validated the positive association of Phospho-YAP^Ser127^ with claudin-2 in xenograft tumors generated by Caki-2 cell and claudin-2 overexpressing cells *(*Fig. 6d).

We further investigated the cellular distribution of YAP in claudin-2 sufficient and deficient cells (MDCKII and MDCKII^TALEN^) using ZO-1 and YAP co-immunofluorescent analysis. As shown in Supplementary Fig. S7 b, we found that YAP expression is significantly more concentrated in nucleus in MDCKII^TALEN^ cells in contrast to MDCKII cells suggesting that the loss of claudin-2 induces nuclear localization of YAP.

To examine further the functional significance of this claudin-2/YAP association, we investigated YAP phosphorylation and nuclear localization in MDCK-II^TALEN^ versus MDCK^CON^ cells. For these studies, we cultured MDCK^CON^ and MDCKII^TALEN^ cells at same density [0.2X10^6^cell/well (6 well plate)] and harvested the samples in a time dependent manner (Supplemntary Fig. S7 c). Immunoblot analysis demonstrated that the expression of P-YAP^Ser127^ increased significantly in a time dependent manner in MDCK^CON^ cells, which was not the case in MDCKII^TALEN^ cells (Fig. 6e). In contrast, total cellular content of YAP protein in MDCK^CON^ cells was not significantly altered while it was significantly upregulated at same time-points in MDCKII^TALEN^ cells. A time-dependent immunofluorescent analysis to determine cellular expression of YAP protein further demonstrated that the nuclear localization of YAP was significantly higher in MDCKII^TALEN^ cells compared to MDCK^CON^ cells (Fig. 6f ). Taken together, these studies suggested a potential role for claudin-2/YAP signaling in regulating PTE cell plasticity and the RCC.

### Claudin-2 binds with YAP by its PDZ-binding motif to regulate its nuclear expression and activation

We further investigated how claudin-2 expression regulates YAP expression/activation by examining the association between claudin-2 and YAP. In this regard, claudin-2 is a transmembrane protein with both, N-terminal and C-terminal in the cell cytosol [40]. The C-terminal of claudin-2 contains a PDZ binding motif that undergoes phosphorylation and mediates interaction with other proteins [35, 41]. We therefore examined if claudin-2 binds with YAP through its PDZ-binding motif by engineering a claudin-2 mutant construct in which the claudin-2 PDZ-binding motif (227**TGYV**230) was deleted. An HA-tag was added to the c-terminal of full-length and mutant claudin-2 constructs (termed as Cldn2^FL/HA^ and Cldn2^ΔPDZ/HA^ respectively) to detect the overexpressed protein. The full-length and PDZ-deleted claudin-2 expression constructs were transfected in both, MDCKII and HK-2 cells. Immunoblot analysis using anti-HA-tag and anti-claudin-2 antibodies confirmed appropriate expression of the newly constructed full-length and mutant proteins (Fig. 6g and Supplementary Fig. S8 a). IP was performed using the cell lysate from both, MDCK-II and HK-2 (control and transfected) cells using anti-HA tag antibody followed by immunoblotting with anti-YAP antibody. As shown in Fig. 6h and Supplementary Fig. S8 b, immunoprecipitation validated that claudin-2 associates with YAP protein through its PDZ-domain, as the association was lost if PDZ-binding motif was deleted. Taken together, these data confirmed that claudin-2 binds with YAP through its PDZ-binding motif.

To further explore pathological significance of above findings, we overexpressed a Cldn2^HA/FL^ and Cldn2^HA/ΔPDZ^ in Caki-2 cells and analyzed the effects on EMT markers and invasion properties. As shown in Fig. 6i, the PDZ-binding mutant claudin-2 expression failed to downregulate expression of the vimentin and fibronectin in Caki-2 cells compared to Cldn2^HA/FL^ transfected cells. Similarly, cell invasion properties were significantly upregulated in Caki-2 cells expressing Cldn2^HA/ΔPDZ^ expression construct versus Cldn2^HA/FL^ transfected cells (Supplementary Fig. S8 c and d). We further investigated effects of the forced expression of constitutive active YAP (mutant) expression construct (YAP^Ser127A^) where serine is swapped with alanine in Caki-2^Cldn2^ cells. Transfection of the wild type YAP protein served as control. Quantitative analysis of the cell invasion indicated that overexpression of the constitutive active YAP in Caki-2^Cldn2^ cells significantly increased invasive ability of these cells despite stable claudin-2 overexpression (Supplementary Fig. S8 e and f). To further ascertain that claudin-2 loss does promotes YAP-signaling, we examined expression of YAP-target genes in claudin-2 knockdown and control HK-2 cell. The qPCR analysis was done for *Kinesin Family Member 14* (*KIF14)*, *Ankyrin Repeat Domain 1(ANKRD1), SRY-Box Transcription Factor 9 (Sox9), and Baculoviral IAP Repeat Containing 5 (Bric5).* As shown in supplementary Fig. S8g, we found that the expression of these YAP-target genes were significantly upregulated in claudin-2 knockdown cells compared to control cells. Taken together, outcome from these studies validated causal role of the claudin-2/YAP signaling in regulating RCC progression (summarized in Fig. 6j).

## Discussion

The concept of EMT has critical considerations in renal homeostasis and diseases, as a switch between an epithelial and mesenchymal phenotype occurs during renal diseases, albeit the extent of this switch and its reversibility are context specific [42]. Our current findings imply a novel role for claudin-2 protein in critical regulation of this switch in renal proximal tubules by helping to maintain the epithelial phenotype, which also appears to be specific to the kidney. In this regard, claudin-2 expression is markedly downregulated in the models of hypoxia/injury and cisplatin injury [10, 43]. Our findings are also consistent with recent studies that claudin-2 KO mice demonstrate exacerbated injury when subjected to hypoxia and claudin-2 expression decreases during renal fibrosis [10, 44] . However, key significance of the overall findings in the current manuscript is that we have identified a previously undescribed causal role of claudin-2 in regulating renal oncogenic growth and progression.

Among claudin family of proteins, claudin-2 is one of the most studied protein due possibly to its role in the paracellular Na + −transport, which partly explains why its renal expression is limited to the PTE, in which there is significant paracellular reabsorption of filtrate [45]. However, PTE is also the primary site for the origin for the RCC [46]. In this regard, studies including ours have demonstrated roles for claudin-2 including in epithelial differentiation, proliferation and migration, albeit in a tissue specific manner. These studies have also revealed that the regulation of renal claudin-2 expression contrasts its regulation in other organs [13, 20, 47]. For example, EGFR-activation promotes claudin-2 expression in intestinal and lung epithelial cells but inhibits it in renal epithelial cells [13, 20, 47]. We found a similar contrasting regulation of intestinal claudin-2 expression versus its renal expression in mice fed a western diet [48]. Notably, obesity increases susceptibility to the RCC [49]. Claudin-2 expression is also upregulated in the colorectal cancer while its expression in RCC is sharply inhibited [20]. This contrasting regulation of claudin-2 led us to thoroughly verify our findings that claudin-2 loss in PTE promotes EMT by using a battery of cell lines, pharmacological and genetic manipulations. However, our conclusion that claudin-2 serves a novel RCC suppressive role is not based solely on the findings from our in vitro studies that the loss of claudin-2 promotes EMT. We also found a significant decrease in claudin-2 expression in RCC and its association with RCC progression and metastasis. Both, analysis of the RNA expression using the TCGA database and protein expression analysis using public database and an independent cohort of the RCC patients supported a significant yet inverse association of claudin-2 expression with RCC progression and metastasis. A causal role for claudin-2 expression in RCC malignancy was further strengthened by the fact that claudin-2 loss associated significantly with poor patient survival. These findings are significant as it may help explain the gender disparity among RCC patients [50]. Of note, claudin-2 is an X-linked gene (https://www.ncbi.nlm. nih.gov/gtr/genes/9075/). Furthermore, as discussed above, renal claudin-2 expression is markedly inhibited in obese mice [48]. Obesity promotes both development and progression of RCC [49]. However, while confirming an essential role for claudin-2 in regulating the tumor cell plasticity and RCC progression, our data also confirms that claudin-2 loss in itself is not oncogenic as claudin-2KO mice do not develop renal tumorigenesis. We therefore postulate that the effects of claudin-2 loss to potentate malignant growth are important only in the context of an oncogenic environment and genetically susceptible hosts. Additional studies are however needed for careful determinations of such context-dependent causal role of claudin-2 and are currently underway in our laboratory.

Our hypothesis that claudin-2 regulates YAP-activation to mediate its effects on PTE cell plasticity and EMT was based on our finding that claudin-2 expression progressively increases in PTE cells with cell density and terminal differentiation. Similar findings in a recent report using MDCK-II cells support this finding [39]. The Hippo/YAP-signaling has been associated with RCC progression [18]. A recent study further demonstrated a role for the EGFR/YAP-signaling in therapeutic responses to the renal diseases [51]. Considering the key role of the cell-cell contact in regulating Hippo/YAP signaling and the fact that EGFR-activation inhibits claudin-2 expression in PTE cells, it appeared logical to investigate whether claudin-2 loss activated YAP-signaling for its oncogenic effects. In this regard, our studies not only confirmed an essential role of YAP-activation in claudin-2 dependent effects on PTE cell plasticity and oncogenic effects but also determined that there is physical partnering between the two proteins. A recent study has reported similar association of claudin-18 with YAP protein in regulating the lung stem cell homeostasis, and tumorigenesis [52]. A key role of the YAP-signaling in claudin-2 dependent effects in our studies was further validated by the fact that expression of a constitutively active YAP construct could overcome the effects of claudin-2 overexpression in Caki-2 cells. We believe that we are the first to report that claudin-2 physically binds with YAP protein to regulate its nuclear expression and activation, and anticipate a broader implication of this finding in renal pathobiology beyond RCC. Of note, claudin-2 has been reported to be part of a large protein complex, and a recent report demonstrated similar binding of claudin-2 protein with proteins implicated in cell polarity, cell-cell adhesion, trafficking and cell multiplication in breast cancer cells [35]. While, our results confirms that claudin-2 binds with YAP through the PDZ-motif, further details of this interaction and regulation of the YAP-phosphorylation is currently underway. In this regard, ZO-1 binds with YAP as well as claudin-2 through its PDZ-motif. We are currently examining whether claudin-2 binds with YAP directly or through ZO-1. Of note, a recent study has reported similar association of claudin-18 with YAP protein in regulating the lung stem cell homeostasis, and tumorigenesis [52]. As noted before, YAP-phosphorylation and nuclear expression are also regulated by the upstream kinases and scaffolding proteins in the Hippo/YAP signaling cascade. Interestingly, our findings that claudin-2 physically interacts with YAP, and the loss of the PDZ-domain of claudin-2 not only disrupts this association but also the tumor-suppressive effects of claudin-2, suggest claudin-2 may regulate YAP-activation through its partnering. However, at this time, we can’t rule out potential effects of claudin-2 expression upon other upstream regulators of the Hippo-signaling, which remains part of our ongoing study.

## Conclusions

In summary, our study highlights a novel kidney specific role of claudin-2 in regulating plasticity and differentiated epithelial phenotype of PTE cells. We believe that our finding that claudin-2/YAP signaling are mediators of PTE cell plasticity may also provide molecular explanation for potential non canonical effects of many cell-cell adhesion proteins in regulating epithelial cell homeostasis. The clinical significance of our finding is considered high in light of the unclear understanding of the molecular regulation of the renal hypoxic injury and fibrosis as an underlying cause for RCC progression.

## Supplementary Information

**Additional file 1.** Supplementary Methods and Material.

**Additional file 2: Fig. S1.** Effects of claudin-2 expression on tumor grade and gender in patients with RCC: (a) Kaplan-Meir analysis to determine patient survival in relation to claudin-2 expression with tumor grade; (b) Kaplan-Meir analysis to determine patient survival in relation to claudin-2 expression with gender. High vs low expression of claudin-2 in tumor grade (P < 0.0001) or patient gender (P < 0.01). Moreover, P=0.028 High vs low (Male), P= 0.013 High vs low (Female) and P= 0.01 High vs low (Female Vs male).

**Additional file 3: Fig. S2.** Constitutive EGFR activation induces EMT in MDCK-II cells and specific loss of claudin-2 expression: Cells stably overexpressing the empty cloning vector (MDCKII^CON^) or a mutant HB-EGF construct that is constitutively secreted into the medium (MDCKII^SHB^) were used. Immunofluorescent analysis of the cellular expression and localization of claudin proteins and ZO-1. Arrows represent membrane tethered and disrupted expression of respective proteins.

**Additional file 4: Fig. S3.** EGFR activation induces EMT in MDCK-II cells and specific loss of claudin-2 expression: (a) Representative phase-contrast images, and Immunofluorescent analysis of the Na+K+ ATPase expression and primary cilium (acetylated-tubulin); (b-c) Immunofluorescent co-localization of Ki-67 and claudin-2 proteins, and Quantitative analysis; (d-e) Representative images of MDCKII^CON^ and MDCKII cells treated with EGF(100ng/ml), subjected to wound-healing (time-course analysis), and Quantitative analysis; (f-g) Anchorage-independent growth (Colony formation assay) in soft agar, and Quantitative analysis; (h-i) Immunoblot analysis of claudin proteins and EMT marker (vimentin and fibronectin), and Densitometric analysis (C= control); (j) Immunofluorescent analysis of the cellular expression and localization of claudin proteins and ZO-1. Arrow represent membrane tethered and disrupted expression of respective proteins. CON represent Control. Data is presented as mean+sem (scale bar =50 μM). Statistical significance was determined by student t test. **P  <  0.01; ***P  <  0.001.

**Additional file 5: Fig. S4.** Hypoxia Induced EMT leads to down regulation claudin-2 in MDCKII and HK2 cells: (a) Representative phase-contrast image and Immunofluorescent analysis of Na+K+ ATPase expression and primary cilium (Acylated tubulin); (b and c) Immunoblot and densitometry of claudins proteins and EMT marker from total lysate of MDCKII cell treated with hypoxic culture environment in time dependent manner. C represent control and hr. represent hours; (d) Representative immunofluorescent image of E-cadherin and vimentin of MDCKII cells exposed to hypoxia; (e) Representative phase-contrast image and immunofluorescence analysis of claudin-2 protein in HK-2 cell culture in hypoxic environment; (f and g) Immunoblot and densitometry analysis of claudins proteins and EMT marker from total lysate of HK2 cell treated with hypoxic culture environment. Data is presented as mean+sem (scale bar =50 μM). Statistical significance was determined by 1-way ANOVA and post hoc Tukey’s test for pairwise comparison. ***P  <  0.001.

**Additional file 6: Fig. S5.** Claudin-2 loss promotes Mesenchymal plasticity in PTE cells and cell proliferation: (a) Phase-contrast image, and (b) immunofluorescent analysis for respective protein (arrow indicated delocalization of expression); (c) Representative immunofluorescent image of E-cadherin and vimentin of MDCKII cells and MDCKII^TALEN^ cells subjected to hypoxia; (d) Representative immunofluorescent image of Ki67 positive cell in MDCKII and MDCKII^TALEN^ cells ; (e) Representative phase-contrast image of the wound-healing; (f and g) Representative immunoblotting and densitometry analysis using tissue lysates from 3D-cultured kidney slices from wild type and claudin-2 knockout (Claudin-2 KO) mice, unchallenged or subjected to hypoxia. CON represent Control. Data is presented as mean+sem. Statistical significance was determined student t test. ***P  <  0.001.

**Additional file 7: Fig. S6.** Claudin-2 overexpression inhibits tumorigenic abilities of Caki-2 cells: (a) Schematic of claudin-2-m-cherry plasmid (pBR-hclaudin-2) expression system dynamics; (b) Fluorescent image of HK-2 cells transfected with pBR-hclaudin-2 plasmid for 48 hours showing GFP protein expression; (c) Fluorescent image of HK-2 cells Co-transfected with pBR-hclaudin-2 plasmid and pCMV-cre plasmid for 48 hours showing GFP and m-cherry expression protein expression; (d) Represent the immunoblotting of claudin-2 from total cell lysate of HK2 cells transfected with pBR-hclaudin-2 plasmid with/without pCMV-Cre plasmid; (e) Representative images of immunofluorescence staining of ki67/claudin-2 in HK-2, Caki-2^CON^ and Caki-2^Cldn2^ cells (f). Representative images of colony formation by HK2, Caki-2^CON^ and Caki-2^Cldn2^ cells. (g) Representative images from matrigel invasion assay showing invaded crystal violet-stained cells in HK-2, Caki-2 and claudin-2 over expressing Caki-2 cells (Caki-2^Cldn2^). Scale bar=50 μM. CON represent Control.

**Additional file 8: Fig. S7.** Claudin-2 interacts with YAP and help maintain membrane expression of the YAP protein: (a) Immunoprecipitation was done using anti-claudin-2 antibody before mass spectrometry (MS) analysis. Lane-1 is protein ladder; Lane-2 is MDCKIITALEN (negative control) Lane-3 MDCKII cell lysate. Arrow indicate selected potential interacting protein with claudin-2; (b) Immunofluorescent analysis of YAP and ZO-1 proteins in exponentially growing MDCKII MDCKII^TALEN^ and MDCKI cells. Arrows indicate localization of the YAP protein; (c) Representative phase-contrast image of MDCKII cells and MDCKII^TALE^N cells subjected to time dependent growth. Data is presented as mean+sem (scale bar =50 μM).

**Additional file 9: Fig. S8.** Claudin-2 PDZ domain interact with YAP and inhibits YAP dependent invasion and regulate the YAP targeted gene expression: (a) Immunoblot analysis of HA-tagged and endogenous claudin-2 expression in HK-2 cell transfected with Full length HA-claudin-2 and PDZ motif deleted claudin-2 plasmid; (b) immunoprecipitation using anti-HA-tag antibody followed by immunoblotting using anti-HA and YAP antibodies from total cell lysate of HK2 cell transfected with Cldn2^HA/Full^ and Cldn2^ΔPDZ/HA^ plasmid constructs; (c and d) effects of Cldn2^FL/HA^ and Cldn2^ΔPDZ/HA^ expression upon cell invasion, and quantitative analysis. Here, *represents comparative analysis between control and Cldn2^FL/HA^ expressing Caki-2 cells and $ represents comparison between Cldn2^FL/HA^ and Cldn2^ΔPDZ/HA^ overexpressing Caki-2 cells; (e and f) Cell invasion and quantitative analysis of Caki-2 cells expressing the Cldn2^FL/HA^ plasmid with or without overexpression of constitutive active YAPS127A expression construct. Here, *represents comparative study between control Cldn2^FL/HA^ overexpressing Caki-2 cells and # represents comparative analysis between cells overexpressing Cldn2^FL/HA^ and Cldn2^FL/HA^+YAP^S127A^ expression plasmid constructs. CON represent Control; (g) qPCR analysis of YAP target genes in HK2 control cell (CON^siRNA^) and claudin-2 knockdown HK2 cells (Cldn2^siRNA^). Data is presented as mean+sem. Statistical significance was determined by student t-test. ***P <  0.001 and ##P < .01 Scale bar=50μM.

**Additional file 10: Supplementary Table S1.** List of reagents, antibodies and kits.

**Additional file 11: Supplementary Table S2.** Primers sequence.

**Additional file 12: Supplementary Table S3.** Selected Potential protein Interacting with Claudin-2.

## Data Availability

The datasets supporting the conclusions of this article are included within the article and its supplementary files.

## Abbreviations

Cldn2: Claudin-2
CON: Control
Co-IP: Co-immunoprecipitation
CPTAC: Clinical proteomic tumor analysis consortium
Ctnnb1: Beta-catenin 1
EGF: Epidermal growth factor
EGFR: Epidermal growth factor receptor
EMT: Epithelial to mesenchymal transition
HA: Hemagglutinin
HB-EGF: Heparin-binding epidermal growth factor
Hr.: Hours
HK-2: Human kidney-2
IF: Immunofluorescence
IHC: Immunohistochemistry
KO: Knockout
MDCK: Madin-darby canine kidney
O_2_: Oxygen
P21: Cyclin dependent kinase inhibitor 1A, encoded by the CDKN1A gene, also known as Cip1
PTE: Proximal tubular epithelium
RCC: Renal clear cell carcinoma
siRNA: Small interfering RNA
TALEN: Transcription activator-like effector nucleases
TCGA: The cancer genome atlas
TMA: Tissue microarray
YAP: Yes associated protein
ZO-1: Zonula occludens-1
