# Supplementary material for: Claudin-2 inhibits renal clear cell carcinoma progression by inhibiting YAP-activation
Source: J Exp Clin Cancer Res. 2021 Feb 23;40:77. doi: 10.1186/s13046-021-01870-5 (PMC7901196; doi:10.1186/s13046-021-01870-5)
Supplement: Supplementary file 1 — Additional file 1. Supplementary Methods and Material. [file 13046_2021_1870_MOESM1_ESM.docx]

**Supplementary Methods and Material**

**Western blot analysis and Immunoprecipitation:** western blot and immunoprecipitation (IP) were done as described previously (1, 2). Briefly, cell or tissue lysates were subjected to sonication before centrifugation at 13,000xg (4°C for 10 min). For immunoprecipitation, lysate was prepared in IP lysis buffer (20 mM Tris HCl pH 8, 137 mM NaCl, 1% Nonidet P-40 (NP-40), 1 mM EDTA). Primary antibody and respective IgG (control) were incubated with the Dynabeads Protein G (Invitrogen #10007D) for at least 1 hour at room temperature. Thereafter, the conjugate was washed with PBS (x2 times) and incubated with 700 mg of protein overnight (4°C). Beads were then washed with Lysis buffer (x 3 times), and samples were prepared in 6x loading dye. Lysates were resolved using SDS-PAGE, and the signal was visualized with horseradish peroxidase-conjugated secondary antibodies using enhanced chemiluminescence (Amersham Biosciences, Piscataway, NJ, USA # A38555).

**Wound healing assay:**Circular wounds were created in respective cell monolayers, and wound closure was followed by phase-contrast imaging (4x) in a time-dependent manner. Quantitative analysis of the wound closer (cell migration) was done by calculating the wound area using Image-J software, as described before (3).

**Immunofluorescence and immunohistochemistry:** Immunostaining were done as previously described (2). In brief, cells were fixed in 4% paraformaldehyde and then washed with phosphate-buffered saline (PBS) containing 50mM NH_4_Cl. Cells were then permeabilized with PBS containing 0.2% Triton X-100 (5 minutes) and incubated with blocking buffer (2% BSA+2.5% normal goat serum) for 1 hour at room temperature followed by incubation with an antigen-specific antibody (in blocking buffer) at 4°C for 16-18 hours. Subsequently, cells were washed with PBS (x5 times) buffer containing 0.1% Triton X-100 and incubated with secondary antibody conjugated with FITC, Cyc-3, or Rhodamine, as required. Images were captured using a Nikon-T2i microscope.

**Invasion assay**: Transwell filters (8.0 μm) were coated overnight with 150 μg/ml collagen type I (Corning # 354236). Cells were suspended at 10,000 cells per well in 24-well filter plates (Falcon; BD) in a serum-free medium. Serum containing medium (0.5%) was utilized as the chemoattractant in the lower chamber. After 48 hours of incubation, cells that had invaded the lower surface of the collagen-coated membrane were fixed with 70% ethanol, stained with crystal violet-blue, and counted in 5 randomly selected fields under a light microscope. Data are expressed as mean ± SD of 3 individual experiments (4).

**Colony formation assay**: A total of 20,000 cells was plated in 1 ml of DMEM with 0.3% low-melting agarose and 10% FBS and overlaid onto 1 ml/35 mm dish of DMEM with 0.5% agarose and 10% FBS. After two weeks, phase-contrast images were taken (×10), and colonies were counted in 5 randomly selected fields. The results represent the mean of at least 3 individual experiments.

**RNA isolation and qRT-PCR analysis: Fold change of mRNA expression** were done as described previously (5). Real-time PCR (qPCR) and gene-specific primers are detailed in Supplementary Table-S2. qPCR reactions were done using 25ng of cDNA/reaction and 2×iQTM SYBR Green Supermix (Bio-Rad, Hercules, CA) and read 25 cycles unless described otherwise.

**Mice tissue processing:**All mice were bred and housed under an Institutional Animal Care, and Use Committee approved protocol. For the microarray experiment, mice were sacrificed at age 15–20 weeks, and the kidneys were flash-frozen in liquid nitrogen. RNA was extracted from the kidney of three individual mice from each group (two from Kras^G12D^/Catnb^Δex3^) and cDNA was created. For immunoblotting and immunohistochemistry, we bred an independent cohort with the same genotypes, harvested the kidneys at 15–20 weeks of age and extracted protein from whole kidneys or fixed and embedded kidneys as described.

**Induction of hypoxia cell culture environment**: Confluent cells were incubated in normal cell culture incubator (CO2 (5%), Oxygen (21%)-Normoxia) or in hypoxia chambers [(CO2 (5%), Nitrogen (94%) and O2 (1%)] for the periods designated in specific studies. Preparation of the hypoxia chamber for desired hypoxic environment was done as per manufacturer (Stem cell Technology) protocol.

**Cell cycle analysis**: MDCKII^CON^ and MDCKII^TALEN^ cells (0.4x10^6^/plate) were seeded in 6-well culture plate. After 24-hour cells of plating, cells were treated with nocodazole (5µg/ml Sigma # M1404-2MG) for 12 hours in serum free medium to synchronize all the cells under investigation. Twelve hours post-nocodazole treatment, cells were cultured in a regular culture medium for an additional 24-hour period. For the final analysis, 1x10^6^ cells were washed with PBS and fixed with 70% ethanol (15 min) at 4oC. Thereafter, cells were washed with cold PBS and stained with Telford reagent (1 ml) for 30 minutes at 4^o^C. The resultant cells were subjected to flow cytometry. Values were calculated as mean+SE from at least three different experiments.

**Xenograft-tumor studies**: All animal experiments were conducted with the approval of the Institutional Animal Care and Use Committee (IACUC) of the University of Nebraska Medical Center. The tumorigenicity of cells under study was assessed using subcutaneous flank inoculation of 1×10^6^ cells in 6-week-old athymic nude mice (n#4/group). Animals were assessed for 3 weeks after subcutaneous implantation of the cancer cells and then sacrificed. Tumor volume was measured using the formula, Tumor volume = 1/2(length × width*2)/2.

**MALDI-TOF Mass Spectroscopy:** Cell lysates were prepared in Immunoprecipitation (IP) lysis buffer (20 mM Tris HCl pH 8, 137 mM NaCl, 1% Nonidet P-40 (NP-40), 1 mM EDTA), and 700 µg protein/sample was incubated with a monoclonal anti-claudin-2 antibody at 4°C for overnight (on Rotating Mixer 3D Shaker). Thereafter, samples were incubated with Protein A/G PLUS-Agarose beads for 4 hours at 4^°C^ , and samples were centrifuged at 8000 rpm for 5 minutes. The resultant palates were washed with IP buffer (8000 rpm x 5 minutes x 3). Pallets were then dissolved in loading buffer and resolved on SDS-PAGE. Gels were then stained with coomassie blue, and appropriate portions of the gels were excised after detaining of the gel for the MALDI-TOF MS analysis, which was done at the UNMC proteomics core facility. The sliced gel was then washed with 100 µl HCPL grad water and further incubated with acetonitrile for 10 minutes. Acetonitrile was then removed by centrifugation, and gels were further incubated with 20mM Tris (2-carboxyethyl) phosphine and ammonium bicarbonate (50mM) (1:10 ratio) at 37°C (30 minutes with continuous shaking). Thereafter, 100μl acetonitrile was added to the tube and incubated for 15 minutes with continuous shaking. Samples were centrifuged and the supernatant was discarded. Resulting pellet was then resuspended in a mixture of Iodoacetamide (200mM; 10μl) and Ammonium bicarbonate (50mM; 90μl) followed by 100 μl of Iodoacetamide solution with continuous shaking in dark for 20 minutes. Samples were centrifuged, the supernatant was discarded and resulting pellet was incubated with acetonitrile (100μl) for 10 minutes. Thereafter trypsin (35μl) was added to the gel and incubated on ice for 30 minutes. Ammonium bicarbonate (25mM; 50μl) was then added to the tube and incubated overnight at 37ºC. After the overnight incubation, samples were transferred to the extraction buffer and incubated for 15 minutes. The sample was then dried in a speedvac. Resultant samples were resuspended in fumaric acid (10μl) and peptide concentration was calculated using a Nano-drop machine.

**Generation of an inducible claudin-2-Mcherry reporter expression construct:** The full-length human claudin-2 cDNA was cloned in a custom cloning construct built upon the pBR plasmid backbone (Supplementary Fig. S6 a). An EGFP reporter gene was placed upstream of claudin-2 cDNA in AgeI and SacII position flanked by loxP-sites. The expression of this EGFP protein was controlled by the Chicken β-promoter. Downstream of the claudin-2 cDNA was placed the m-cherry gene and its expression was regulated by a bi-cistronic promoter. The external expression of a Cre protein (by transfection of a Cre expression plasmid construct) led to the Cre/Lox excision of the EGFP gene and homologous recombination between the loxP sites resulting in the expression of claudin-2 protein and m-cherry protein as C-terminal non-fused reporter protein. HK2 cell transfected with pBR-hclaudin-2 plasmid with and without pCMV-cre plasmid. Forty-eight hours post-transfection, fluorescent images were taken. Cells transfected with only pBR-hclaudin-2 plasmid alone showed expression of the green florescent protein (GFP) and cells co-transfected with pBR-hclaudin-2 and pCMV-cre plasmids showed GFP and m-cherry co-expression (Supplementary Fig. S6 b and c). Immunoblot analysis using total cells lysate from pBR-hclaudin-2 and p-CMV-cre co-transfected cells represented claudin-2 overexpression and cells expressing GFP alone served as control (Supplementary Figure S6 d)

**Reference:**

1. Singh AB, and Harris RC. Epidermal growth factor receptor activation differentially regulates claudin expression and enhances transepithelial resistance in Madin-Darby canine kidney cells. J Biol Chem. 2004;279(5):3543-52.

2. Ahmad R, Kumar B, Pan K, Dhawan P, and Singh AB. HDAC-4 regulates claudin-2 expression in EGFR-ERK1/2 dependent manner to regulate colonic epithelial cell differentiation. Oncotarget. 2017;8(50):87718-36.

3. Bhat AA, Ahmad R, Uppada SB, Singh AB, and Dhawan P. Claudin-1 promotes TNF-alpha-induced epithelial-mesenchymal transition and migration in colorectal adenocarcinoma cells. Exp Cell Res. 2016;349(1):119-27.

4. Dhawan P, Singh AB, Deane NG, No Y, Shiou SR, Schmidt C, et al. Claudin-1 regulates cellular transformation and metastatic behavior in colon cancer. J Clin Invest. 2005;115(7):1765-76.

5. Ahmad R, Chaturvedi R, Olivares-Villagomez D, Habib T, Asim M, Shivesh P, et al. Targeted colonic claudin-2 expression renders resistance to epithelial injury, induces immune suppression, and protects from colitis. Mucosal Immunol. 2014;7(6):1340-53.
