# Supplementary figures and images for: Claudin-2 inhibits renal clear cell carcinoma progression by inhibiting YAP-activation
Source: J Exp Clin Cancer Res. 2021 Feb 23;40:77. doi: 10.1186/s13046-021-01870-5 (PMC7901196; doi:10.1186/s13046-021-01870-5)

Fig.S 1

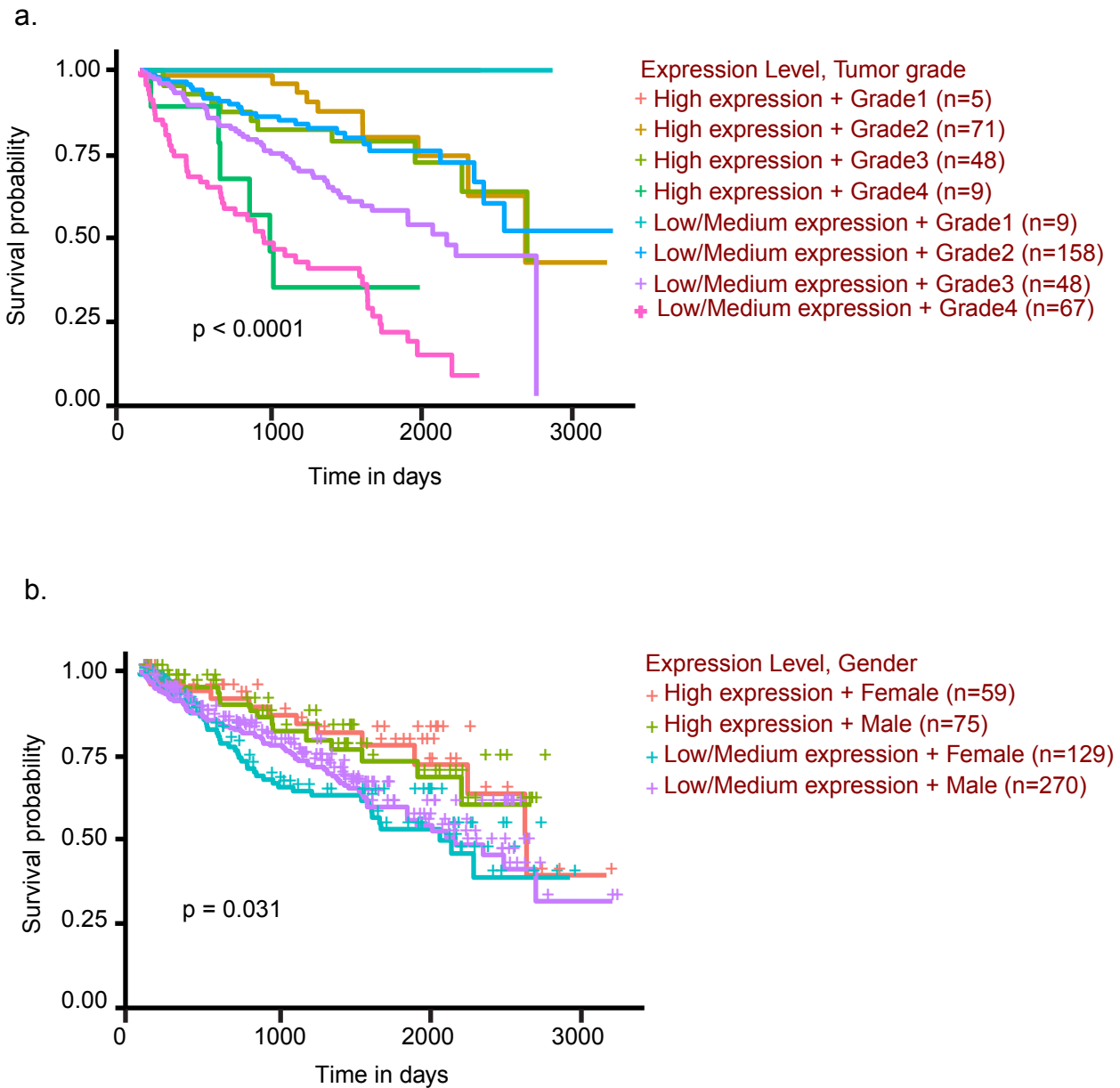

Supplement: Supplementary file 2 — Additional file 2: Fig. S1. Effects of claudin-2 expression on tumor grade and gender in patients with RCC: (a) Kaplan-Meir analysis to determine patient survival in relation to claudin-2 expression with tumor grade; (b) Kaplan-Meir analysis to determine patient survival in relation to claudin-2 expression with gender. High vs low expression of claudin-2 in tumor grade (P < 0.0001) or patient gender (P < 0.01). Moreover, P=0.028 High vs low (Male), P= 0.013 High vs low (Female) and P= 0.01 High vs low (Female Vs male). [file 13046_2021_1870_MOESM2_ESM.pdf]

Fig.S 2

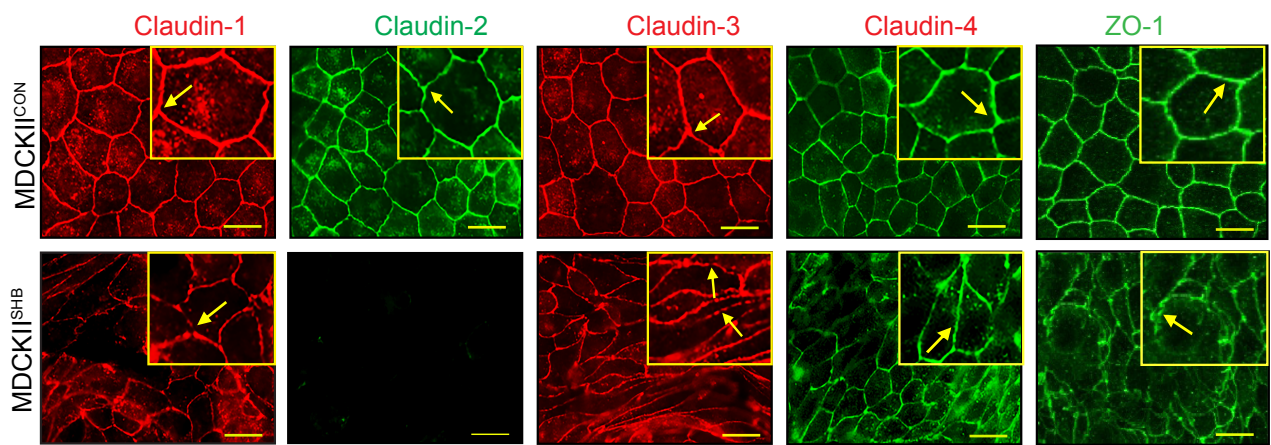

Supplement: Supplementary file 3 — Additional file 3: Fig. S2. Constitutive EGFR activation induces EMT in MDCK-II cells and specific loss of claudin-2 expression: Cells stably overexpressing the empty cloning vector (MDCKIICON) or a mutant HB-EGF construct that is constitutively secreted into the medium (MDCKIISHB) were used. Immunofluorescent analysis of the cellular expression and localization of claudin proteins and ZO-1. Arrows represent membrane tethered and disrupted expression of respective proteins. [file 13046_2021_1870_MOESM3_ESM.pdf]

Fig.S 3

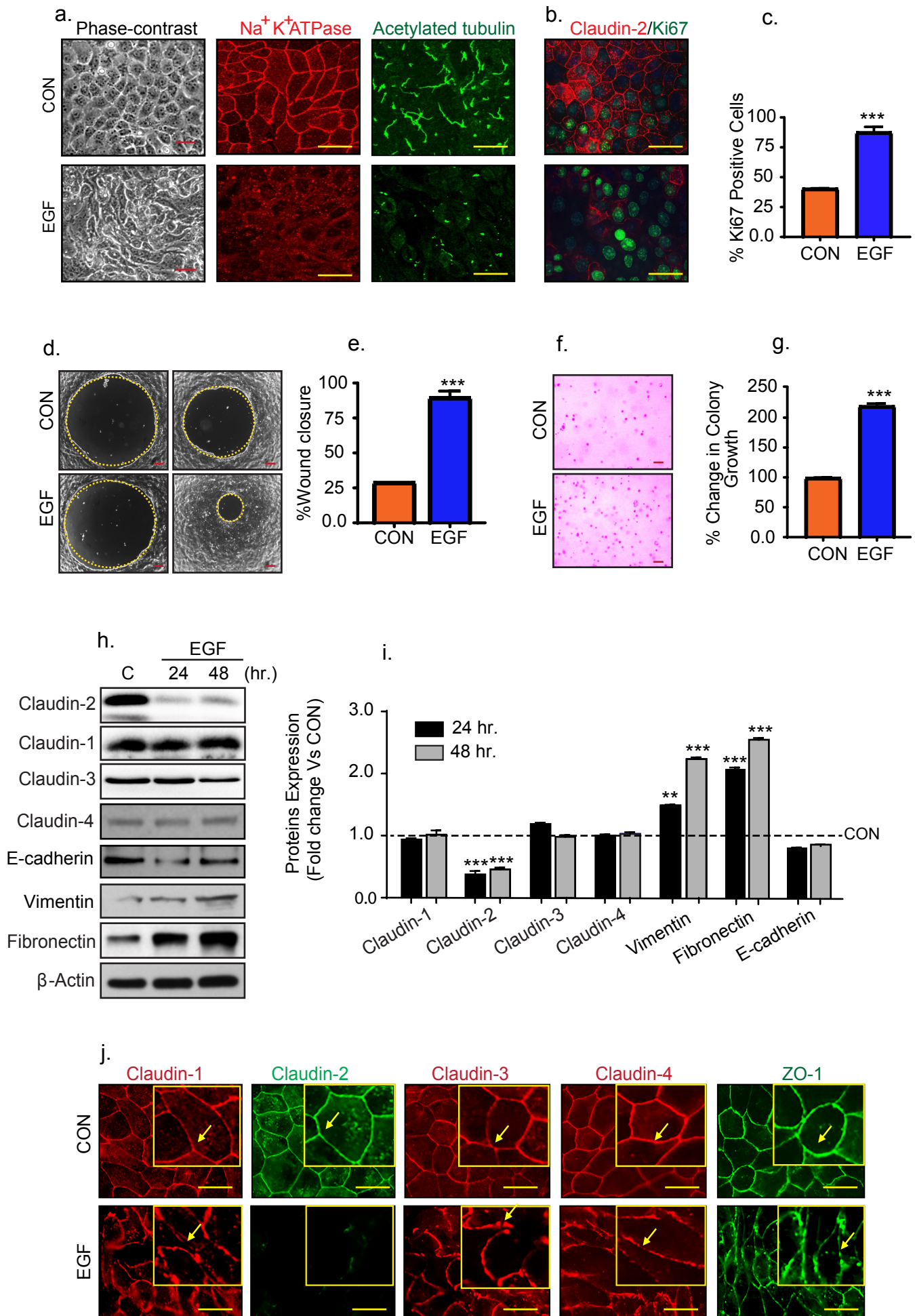

Supplement: Supplementary file 4 — Additional file 4: Fig. S3. EGFR activation induces EMT in MDCK-II cells and specific loss of claudin-2 expression: (a) Representative phase-contrast images, and Immunofluorescent analysis of the Na+K+ ATPase expression and primary cilium (acetylated-tubulin); (b-c) Immunofluorescent co-localization of Ki-67 and claudin-2 proteins, and Quantitative analysis; (d-e) Representative images of MDCKIICON and MDCKII cells treated with EGF(100ng/ml), subjected to wound-healing (time-course analysis), and Quantitative analysis; (f-g) Anchorage-independent growth (Colony formation assay) in soft agar, and Quantitative analysis; (h-i) Immunoblot analysis of claudin proteins and EMT marker (vimentin and fibronectin), and Densitometric analysis (C= control); (j) Immunofluorescent analysis of the cellular expression and localization of claudin proteins and ZO-1. Arrow represent membrane tethered and disrupted expression of respective proteins. CON represent Control. Data is presented as mean+sem (scale bar =50 μM). Statistical significance was determined by student t test. **P < 0.01; ***P < 0.001. [file 13046_2021_1870_MOESM4_ESM.pdf]

Fig.S 4

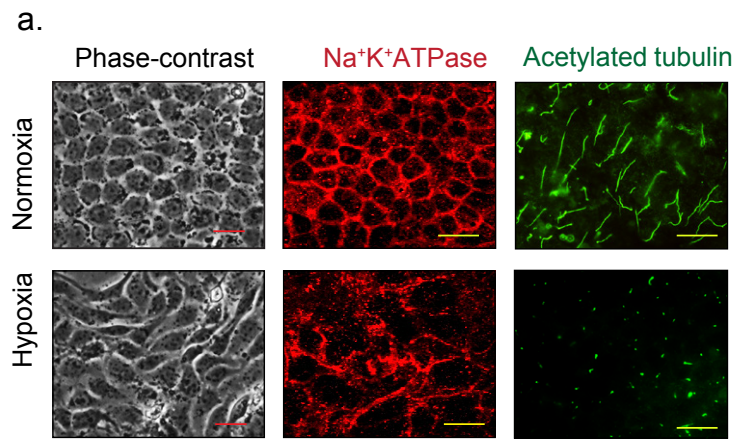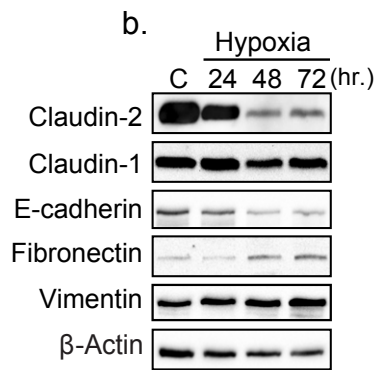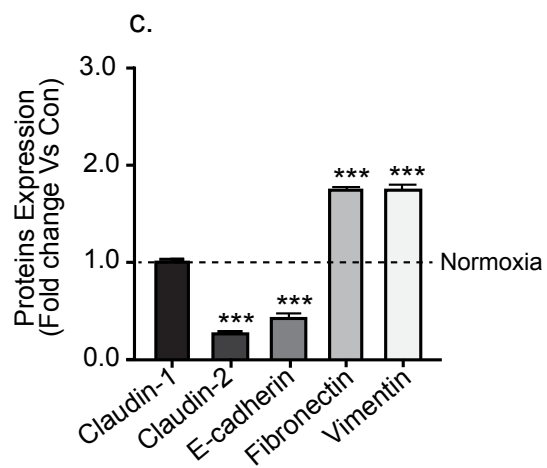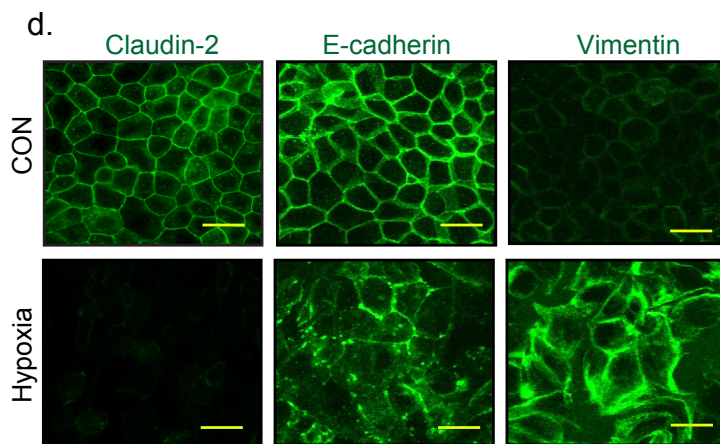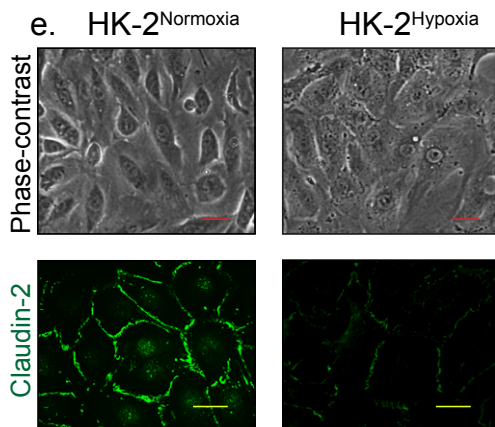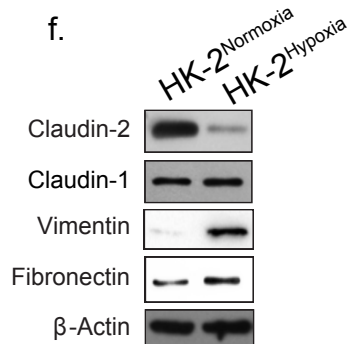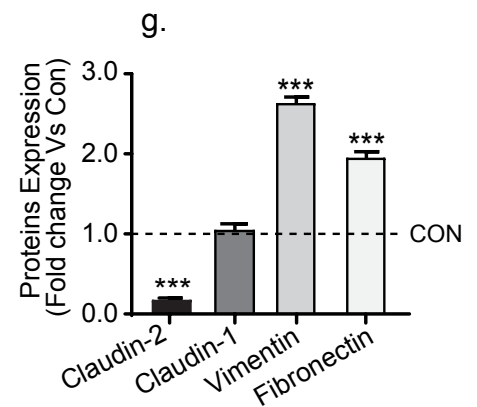

Supplement: Supplementary file 5 — Additional file 5: Fig. S4. Hypoxia Induced EMT leads to down regulation claudin-2 in MDCKII and HK2 cells: (a) Representative phase-contrast image and Immunofluorescent analysis of Na+K+ ATPase expression and primary cilium (Acylated tubulin); (b and c) Immunoblot and densitometry of claudins proteins and EMT marker from total lysate of MDCKII cell treated with hypoxic culture environment in time dependent manner. C represent control and hr. represent hours; (d) Representative immunofluorescent image of E-cadherin and vimentin of MDCKII cells exposed to hypoxia; (e) Representative phase-contrast image and immunofluorescence analysis of claudin-2 protein in HK-2 cell culture in hypoxic environment; (f and g) Immunoblot and densitometry analysis of claudins proteins and EMT marker from total lysate of HK2 cell treated with hypoxic culture environment. Data is presented as mean+sem (scale bar =50 μM). Statistical significance was determined by 1-way ANOVA and post hoc Tukey’s test for pairwise comparison. ***P < 0.001. [file 13046_2021_1870_MOESM5_ESM.pdf]

Fig.S 5

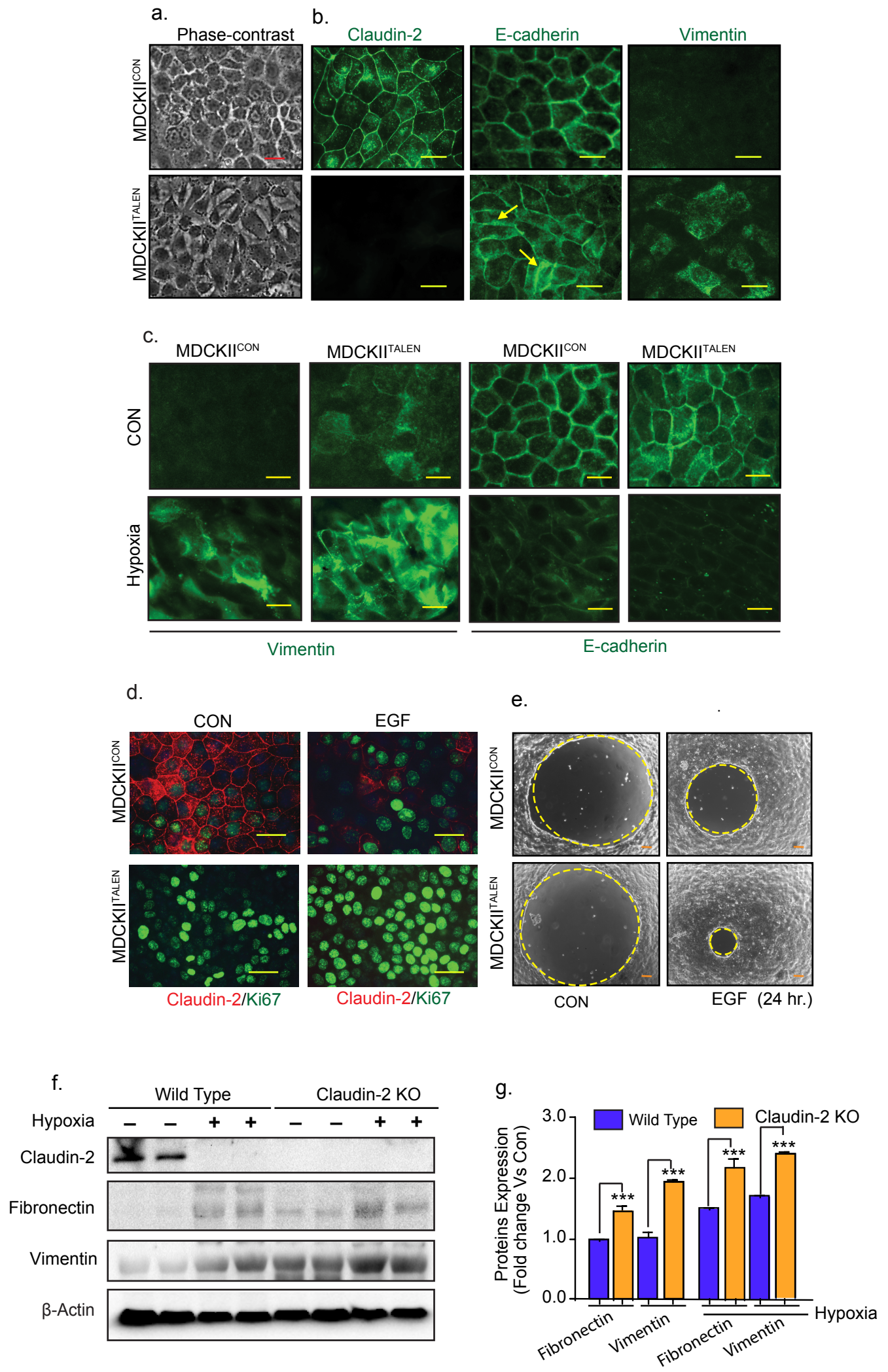

Supplement: Supplementary file 6 — Additional file 6: Fig. S5. Claudin-2 loss promotes Mesenchymal plasticity in PTE cells and cell proliferation: (a) Phase-contrast image, and (b) immunofluorescent analysis for respective protein (arrow indicated delocalization of expression); (c) Representative immunofluorescent image of E-cadherin and vimentin of MDCKII cells and MDCKIITALEN cells subjected to hypoxia; (d) Representative immunofluorescent image of Ki67 positive cell in MDCKII and MDCKIITALEN cells ; (e) Representative phase-contrast image of the wound-healing; (f and g) Representative immunoblotting and densitometry analysis using tissue lysates from 3D-cultured kidney slices from wild type and claudin-2 knockout (Claudin-2 KO) mice, unchallenged or subjected to hypoxia. CON represent Control. Data is presented as mean+sem. Statistical significance was determined student t test. ***P < 0.001. [file 13046_2021_1870_MOESM6_ESM.pdf]

Fig.S 7

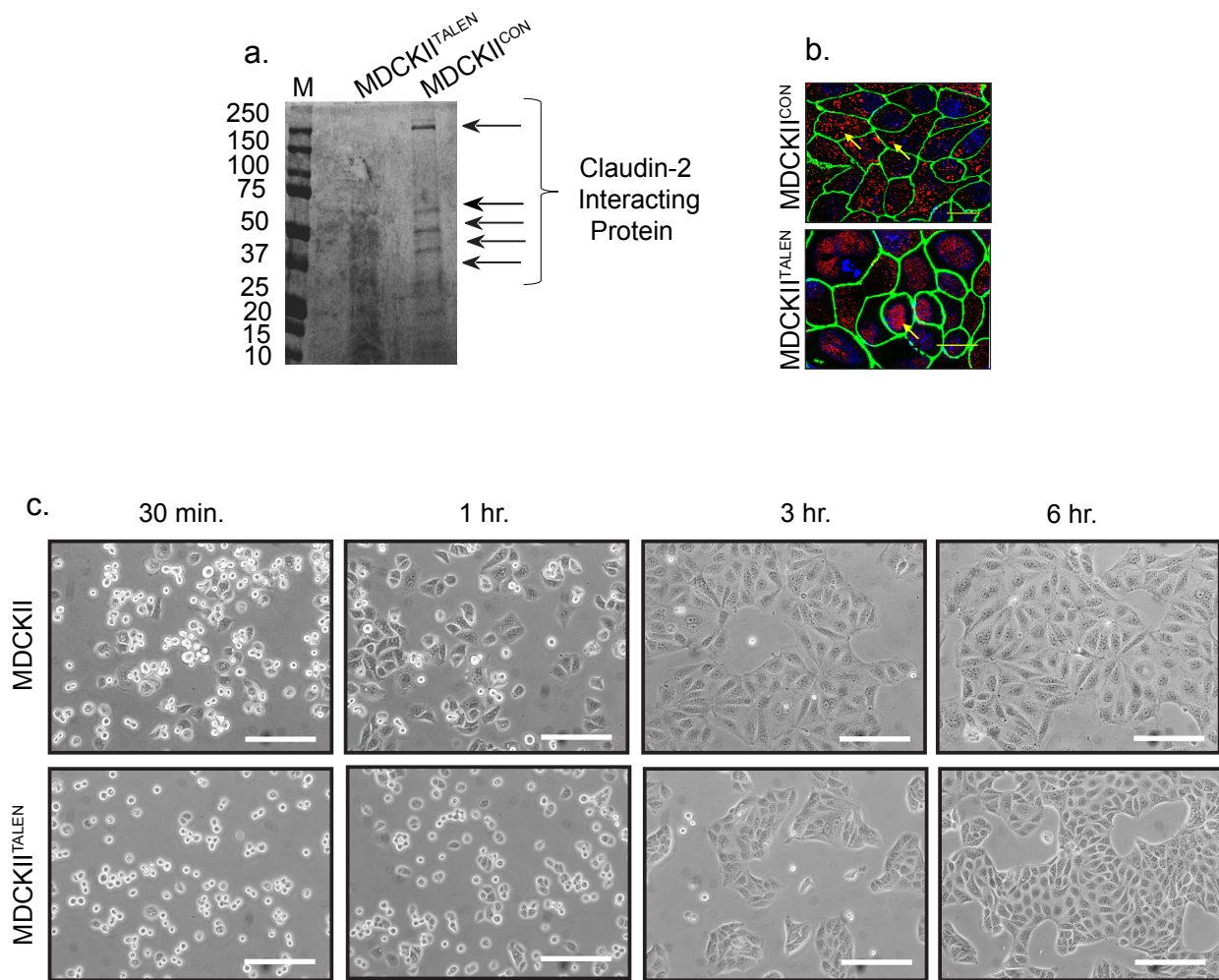

Supplement: Supplementary file 8 — Additional file 8: Fig. S7. Claudin-2 interacts with YAP and help maintain membrane expression of the YAP protein: (a) Immunoprecipitation was done using anti-claudin-2 antibody before mass spectrometry (MS) analysis. Lane-1 is protein ladder; Lane-2 is MDCKIITALEN (negative control) Lane-3 MDCKII cell lysate. Arrow indicate selected potential interacting protein with claudin-2; (b) Immunofluorescent analysis of YAP and ZO-1 proteins in exponentially growing MDCKII MDCKIITALEN and MDCKI cells. Arrows indicate localization of the YAP protein; (c) Representative phase-contrast image of MDCKII cells and MDCKIITALEN cells subjected to time dependent growth. Data is presented as mean+sem (scale bar =50 μM). [file 13046_2021_1870_MOESM8_ESM.pdf]

Fig.S 8

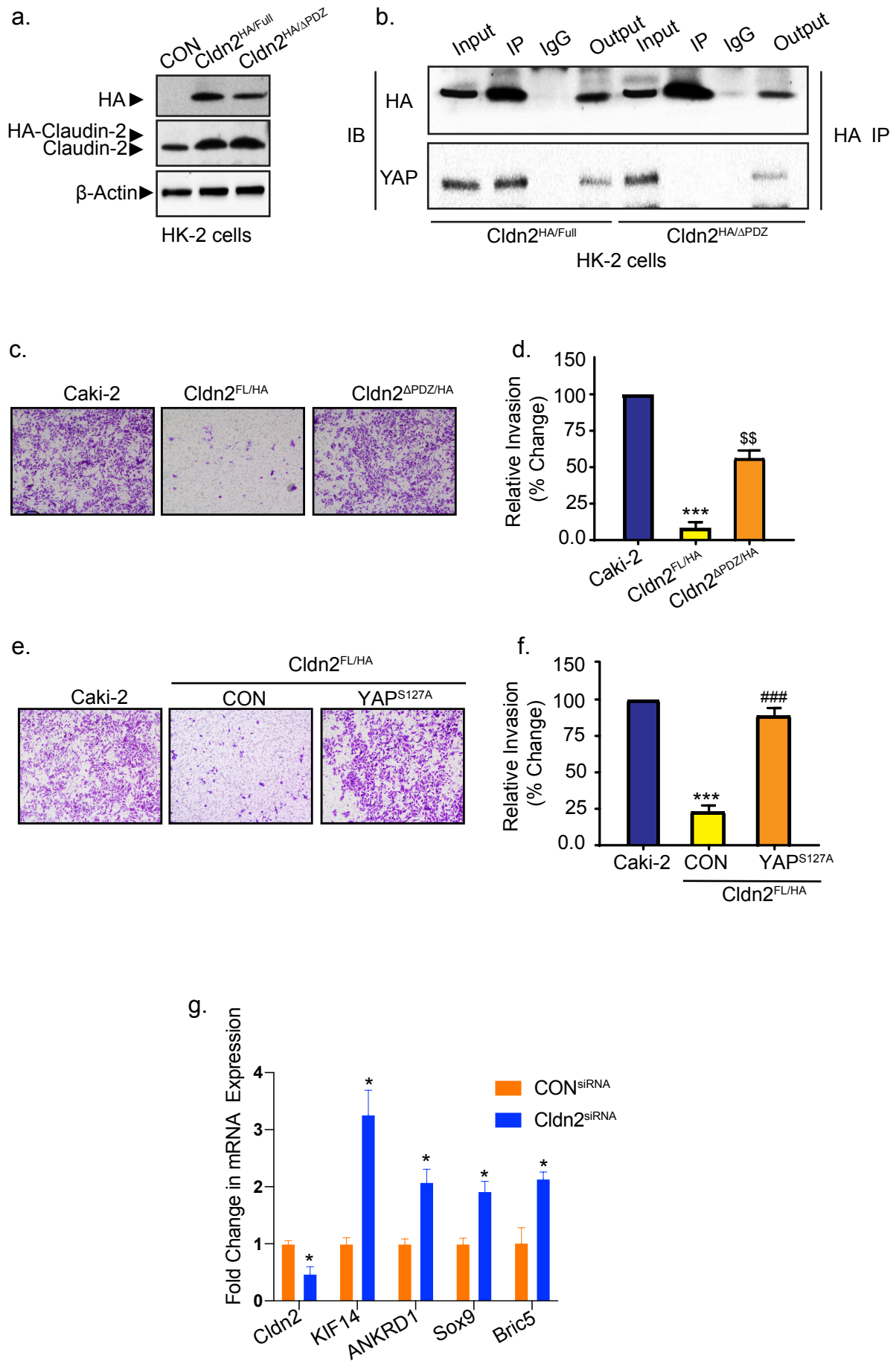

Supplement: Supplementary file 9 — Additional file 9: Fig. S8. Claudin-2 PDZ domain interact with YAP and inhibits YAP dependent invasion and regulate the YAP targeted gene expression: (a) Immunoblot analysis of HA-tagged and endogenous claudin-2 expression in HK-2 cell transfected with Full length HA-claudin-2 and PDZ motif deleted claudin-2 plasmid; (b) immunoprecipitation using anti-HA-tag antibody followed by immunoblotting using anti-HA and YAP antibodies from total cell lysate of HK2 cell transfected with Cldn2HA/Full and Cldn2ΔPDZ/HA plasmid constructs; (c and d) effects of Cldn2FL/HA and Cldn2ΔPDZ/HA expression upon cell invasion, and quantitative analysis. Here, *represents comparative analysis between control and Cldn2FL/HA expressing Caki-2 cells and $ represents comparison between Cldn2FL/HA and Cldn2ΔPDZ/HA overexpressing Caki-2 cells; (e and f) Cell invasion and quantitative analysis of Caki-2 cells expressing the Cldn2FL/HA plasmid with or without overexpression of constitutive active YAPS127A expression construct. Here, *represents comparative study between control Cldn2FL/HA overexpressing Caki-2 cells and # represents comparative analysis between cells overexpressing Cldn2FL/HA and Cldn2FL/HA+YAPS127A expression plasmid constructs. CON represent Control; (g) qPCR analysis of YAP target genes in HK2 control cell (CONsiRNA) and claudin-2 knockdown HK2 cells (Cldn2siRNA). Data is presented as mean+sem. Statistical significance was determined by student t-test. ***P < 0.001 and ##P < .01 Scale bar=50μM. [file 13046_2021_1870_MOESM9_ESM.pdf]
