# Supplementary material for: Claudin-2 inhibits renal clear cell carcinoma progression by inhibiting YAP-activation
Source: J Exp Clin Cancer Res. 2021 Feb 23;40:77. doi: 10.1186/s13046-021-01870-5 (PMC7901196; doi:10.1186/s13046-021-01870-5)
Supplement: Supplementary file 7 — Additional file 7: Fig. S6. Claudin-2 overexpression inhibits tumorigenic abilities of Caki-2 cells: (a) Schematic of claudin-2-m-cherry plasmid (pBR-hclaudin-2) expression system dynamics; (b) Fluorescent image of HK-2 cells transfected with pBR-hclaudin-2 plasmid for 48 hours showing GFP protein expression; (c) Fluorescent image of HK-2 cells Co-transfected with pBR-hclaudin-2 plasmid and pCMV-cre plasmid for 48 hours showing GFP and m-cherry expression protein expression; (d) Represent the immunoblotting of claudin-2 from total cell lysate of HK2 cells transfected with pBR-hclaudin-2 plasmid with/without pCMV-Cre plasmid; (e) Representative images of immunofluorescence staining of ki67/claudin-2 in HK-2, Caki-2CON and Caki-2Cldn2 cells (f). Representative images of colony formation by HK2, Caki-2CON and Caki-2Cldn2 cells. (g) Representative images from matrigel invasion assay showing invaded crystal violet-stained cells in HK-2, Caki-2 and claudin-2 over expressing Caki-2 cells (Caki-2Cldn2). Scale bar=50 μM. CON represent Control. [file 13046_2021_1870_MOESM7_ESM.pdf]

Fig.S 6

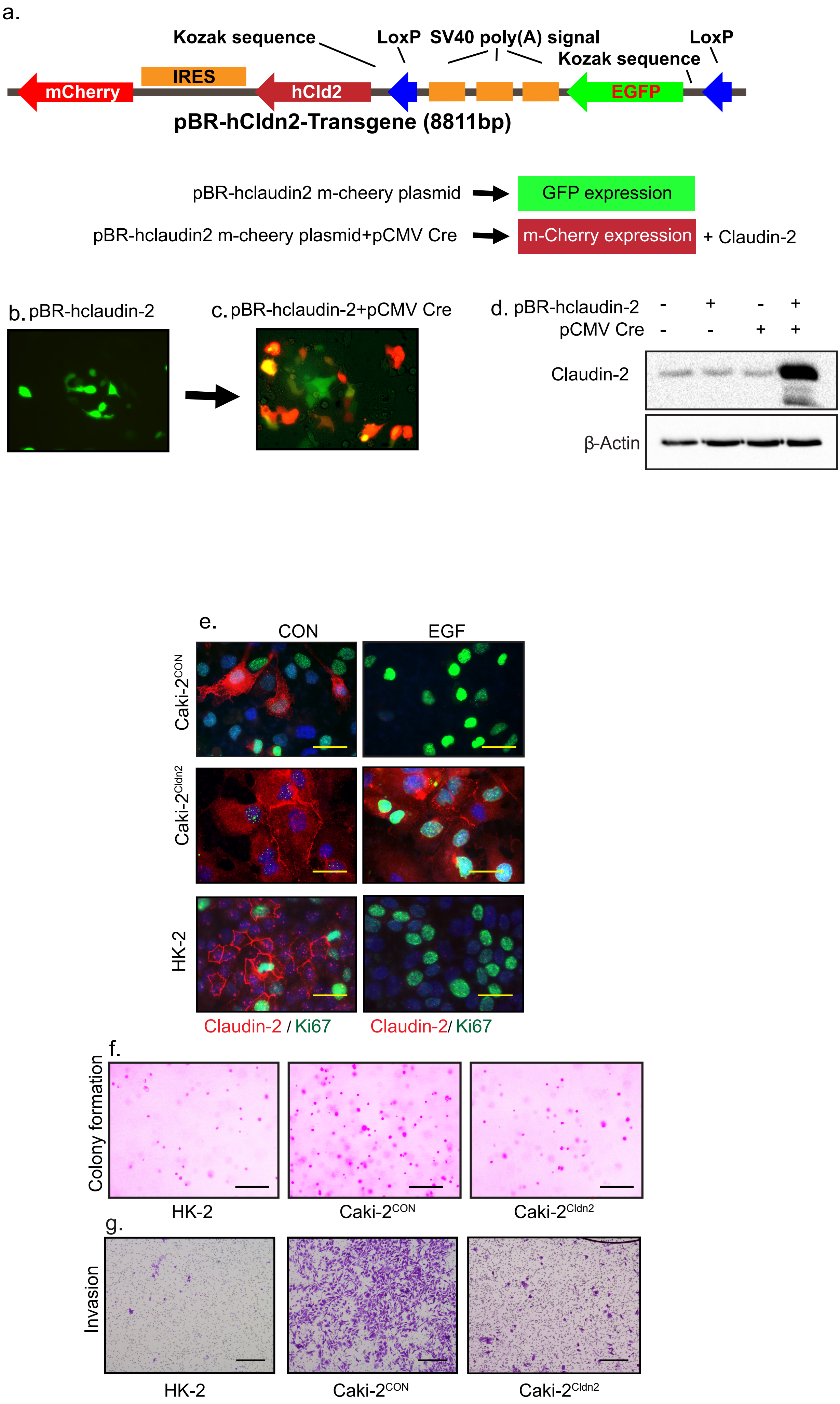
a.
Kozak sequence
LoxP
SV40 poly(A) signal
LoxP
Kozak sequence
IRES
mCherry
hCld2
EGFP
pBR-hCldn2-Transgene (8811bp)
pBR-hclaudin2 m-cheery plasmid
GFP expression
pBR-hclaudin2 m-cheery plasmid+pCMV Cre
m-Cherry expression
+ Claudin-2
b. pBR-hclaudin-2
c. pBR-hclaudin-2+pCMV Cre
d. pBR-hclaudin-2 - + - +
pCMV Cre - - + +
Claudin-2
β-Actin
e.
CON
EGF
Caki-2CON
Caki-2Cldn2
HK-2
Claudin-2 / Ki67
Claudin-2/ Ki67
f.
Colony formation
HK-2
Caki-2CON
Caki-2Cldn2
g.
Invasion
HK-2
Caki-2CON
Caki-2Cldn2
