# Supplementary material for: Claudin-2 inhibits renal clear cell carcinoma progression by inhibiting YAP-activation
Source: J Exp Clin Cancer Res. 2021 Feb 23;40:77. doi: 10.1186/s13046-021-01870-5 (PMC7901196; doi:10.1186/s13046-021-01870-5)
Supplement: Supplementary file 10 — Additional file 10: Supplementary Table S1. List of reagents, antibodies and kits. [file 13046_2021_1870_MOESM10_ESM.docx]

**Supplementary Table S1.**

List of reagents, antibodies and kits.

| **S.N** | **Antibody** | **Company** | **Catalog Number** |
| --- | --- | --- | --- |
| 1 | Claudin 2 Mouse monoclonal antibody (12H12) | Invitrogen | 32-5600 |
| 2 | Claudin 1 Mouse monoclonal antibody (2H10D10) | Invitrogen | 37-4900 |
| 3 | Claudin3 Rabbit Polyclonal Antibody | Invitrogen | PA5-16867 |
| 4 | Claudin 4 Mouse monoclonal Antibody (3E2C1) | Invitrogen | 32-9400 |
| 5 | E-cadherin Mouse monoclonal antibody | BD biosciences | 610181 |
| 6 | Fibronectin Mouse monoclonal antibody | BD biosciences | 610077 |
| 7 | P21 Mouse monoclonal antibody | Santa Cruz Biotechnology | Sc6246 |
| 8 | Phospho-YAP(Ser127) Rabbit monoclonal antibody | Cell signaling Technology | 13008 |
| 9 | YAP Rabbit monoclonal antibody | Cell signaling Technology | 14074 |
| 10 | Alexa Fluor 488 Phalloidin | Invitrogen | A12379 |
| 11 | ZO-1 Mouse monoclonal antibody | Invitrogen | 339100 |
| 13 | ZO-1 Rabbit monoclonal antibody | Invitrogen | 61-7300 |
| 14 | HA-Tag Rabbit monoclonal antibody | Cell signaling Technology | 2367S |
| 15 | Anti-Acetylated Mouse monoclonal Tubulin antibody | Sigma | T7451 |
| 16 | Na,K-ATPase α1 (D4Y7E) Rabbit monoclonal antibody | Cell signaling Technology | 23565 |
| 17 | Vimentin Mouse monoclonal antibody | Invitrogen | MA3-745 |
| 18 | jetPRIME transfection Reagent | Polyplus transfection | 114-07 |
| 19 | Claudin-1 Rabbit polyclonal antibody | Invitrogen | 71-7800 |
| 20 | Ki67 Rabbit monoclonal antibody | Cell signaling Technology | 12202 |
| 21 | VECTASTAIN Elite ABC Universal PLUS Kit | Vector Laboratory | PK-8200 |
| 22 | Claudin-2 siRNA | Thermo fisher | 4392420 |
| 23 | Hippo Signaling antibody Sampler Kit | Cell signaling Technology | 8579 |
| 24 | Corning Matrigel Matrix | Corning | 356255, 354236 |
