# Supplementary material for: Claudin-2 inhibits renal clear cell carcinoma progression by inhibiting YAP-activation
Source: J Exp Clin Cancer Res. 2021 Feb 23;40:77. doi: 10.1186/s13046-021-01870-5 (PMC7901196; doi:10.1186/s13046-021-01870-5)
Supplement: Supplementary file 11 — Additional file 11: Supplementary Table S2. Primers sequence. [file 13046_2021_1870_MOESM11_ESM.docx]

**Supplementary Table S2.**

Primers sequence

| **S.N.** | **Primer Name** | **5’---------------Sequence--------------3’** |
| --- | --- | --- |
| 1 | M.Claudin-2. FP | GATTGGAGAGGCTCTGTACTTG |
| 2 | M.Claudin-2. RP | TAGTTGGTACGATTGCCCTG |
| 3 | M.Claudin-14. FP | AGGCTGAATGACTACGTGTG |
| 4 | M.Claudin-14. RP | CTCCTTCCCTGTGTTCCG |
| 5 | H.ANKRD1 FP | GCCTACGTTTCTGAAGGCTG |
| 6 | *H.ANKRD1* RP | GTGGATTCAAGCATATCACGGAA |
| 7 | *KIF14*.HF | CCGACATTACAGATGCACCA |
| 8 | *KIF14.*HR | CTTCATTCCTAAGCCTACACC |
| 9 | *SOX9*.HF | TGCAGGAGGAGAAGAGAAGG |
| 10 | *SOX9*.HR | GTGGCCAGTTCACAGCTGC |
| 11 | *Bric5*.FP | GACCACCGCATCTCTACATTC |
| 12 | *Bric5*.RP | TGCTTTTTATGTTCCTCTATGGG |
