# Supplementary material for: Claudin-2 inhibits renal clear cell carcinoma progression by inhibiting YAP-activation
Source: J Exp Clin Cancer Res. 2021 Feb 23;40:77. doi: 10.1186/s13046-021-01870-5 (PMC7901196; doi:10.1186/s13046-021-01870-5)
Supplement: Supplementary file 12 — Additional file 12: Supplementary Table S3. Selected Potential protein Interacting with Claudin-2. [file 13046_2021_1870_MOESM12_ESM.docx]

**Supplementary Table S3.**

**Selected Potential protein Interacting with Claudin-2**

| S.N. | Protein | Probability legend |
| --- | --- | --- |
| 1 | Claudin-2 | Over 95% |
| 2 | Tight junction protein ZO1 | Over 95% |
| 3 | Ubiquitin carboxyl-terminal hydrolase | Over 95% |
| 4 | Cingulin | Over 95% |
| 5 | Caveolin-1 | Over 95% |
| 6 | Yes-associated protein | Over 95% |
